# Osteocyte Network; a Negative Regulatory System for Bone Mass Augmented by the Induction of Rankl in Osteoblasts and Sost in Osteocytes at Unloading

**DOI:** 10.1371/journal.pone.0040143

**Published:** 2012-06-29

**Authors:** Takeshi Moriishi, Ryo Fukuyama, Masako Ito, Toshihiro Miyazaki, Takafumi Maeno, Yosuke Kawai, Hisato Komori, Toshihisa Komori

**Affiliations:** 1 Department of Cell Biology, Nagasaki University Graduate School of Biomedical Sciences, Nagasaki, Japan; 2 Laboratory of Pharmacology, Hiroshima International University, Kure, Japan; 3 Department of Radiology and Radiation Biology, Nagasaki University Graduate School of Biomedical Sciences, Nagasaki, Japan; 4 Department of Orthopedic Surgery, Osaka City University Graduate School of Medicine, Osaka, Japan; 5 Department of Regenerative Oral Surgery, Nagasaki University Graduate School of Biomedical Sciences, Nagasaki, Japan; Cincinnati Children’s Hospital Medical Center, United States of America

## Abstract

Reduced mechanical stress is a major cause of osteoporosis in the elderly, and the osteocyte network, which comprises a communication system through processes and canaliculi throughout bone, is thought to be a mechanosensor and mechanotransduction system; however, the functions of osteocytes are still controversial and remain to be clarified. Unexpectedly, we found that overexpression of *BCL2* in osteoblasts eventually caused osteocyte apoptosis. Osteoblast and osteoclast differentiation were unaffected by *BCL2* transgene in vitro. However, the cortical bone mass increased due to enhanced osteoblast function and suppressed osteoclastogenesis at 4 months of age, when the frequency of TUNEL-positive lacunae reached 75%. In the unloaded condition, the trabecular bone mass decreased in both wild-type and *BCL2* transgenic mice at 6 weeks of age, while it decreased due to impaired osteoblast function and enhanced osteoclastogenesis in wild-type mice but not in *BCL*2 transgenic mice at 4 months of age. *Rankl* and *Opg* were highly expressed in osteocytes, but *Rankl* expression in osteoblasts but not in osteocytes was increased at unloading in wild-type mice but not in *BCL*2 transgenic mice at 4 months of age. Sost was locally induced at unloading in wild-type mice but not in *BCL*2 transgenic mice, and the dissemination of Sost was severely interrupted in *BCL*2 transgenic mice, showing the severely impaired osteocyte network. These findings indicate that the osteocyte network is required for the upregulation of *Rankl* in osteoblasts and Sost in osteocytes in the unloaded condition. These findings suggest that the osteocyte network negatively regulate bone mass by inhibiting osteoblast function and activating osteoclastogenesis, and these functions are augmented in the unloaded condition at least partly through the upregulation of *Rankl* expression in osteoblasts and that of Sost in osteocytes, although it cannot be excluded that low *BCL2* transgene expression in osteoblasts contributed to the enhanced osteoblast function.

## Introduction

Bone tissue is able to adapt its mass and three-dimensional structure to the prevailing mechanical usage to achieve higher load-bearing efficiency [1]. The lacunocanalicular network formed by osteocytes is thought to be an ideal mechanosensory system and suitable for mechanotransduction, by which mechanical energy is converted into electrical and/or biochemical signals [2], [3], [4], [5], [6], [7]; however, the function of the osteocyte network in the regulation of bone mass remains to be clarified.

The function of osteocytes in bone formation is controversial. Osteocytes have been considered to activate bone formation, because osteocytes induced anabolic factors, such as prostaglandin E_2_ (PGE_2_), prostaglandin I_2_ (PGI_2_), nitric oxide (NO), cyclooxygenase-2 (COX-2), and endothelial nitric oxide synthase (ecNOS), after application of mechanical stimuli in vitro [5] and bone formation was severely inhibited after osteocyte ablation [8]. However, Marotti et al. theorized that osteocytes inhibit osteoblasts by means of inhibitory signals transmitted via gap junctions and recruit selected osteoblasts to the osteocyte lineage [9]. In accordance with this theory, osteocyte density and bone formation rate were inversely related [10], [11]. Further, Sclerostin, the *SOST* gene protein product, is specifically expressed in osteocytes and inhibits osteoblast function and bone formation by antagonizing canonical Wnt signaling through the binding to Wnt co-receptor low density lipoprotein receptor-related protein (LRP) 5 and LRP6, and *Sost*-deficient mice are resistant to bone loss at unloading [12], [13], [14], [15], [16], [17], [18], [19], [20].

**Table 1:** Bone histomorphometric analysis of trabecular bone at 10 weeks and 4 months of age.

genotype (age)	BV/TV	O.Th	N.Ob/B.Pm	N.OC/B.Pm	ES/BS	Osteocyte/Ar	MAR	MS/BS	BFR/BS
	(%)	(µm)	(/100 mm)	(/100 mm)	(%)	(/mm^2^)	(µm/day)	(%)	(mm^3^/mm^2^/year)
wt (10w)	16.4±7.2	2.56±0.37	1715±397.7	296.4±64.4	9.7±2.8	451.7±67.7	1.77±0.33	28.3±4.8	0.18±0.06
tg (10w)	14.5±2.0	2.54±0.69	3472±1020.5*	262.8±103.9	9.0±3.2	850.3±153.6**	1.32±0.32	45.0±11.2	0.20±0.07
wt (4m)	18.3±4.3	1.32±0.20	599±256.5	147.6±56.7	4.9±1.8	405.1±62.5	1.33±0.30	12.4±4.0	0.06±0.02
tg (4m)	24.8±13.7^#^	1.87±0.83^#^	448±161.5	166.4±19.9	5.6±0.6	314.5±67.5^#^	1.71±0.30^#^	36.2±16.4^##^	0.24±0.12^##^

MS: mineralizing surface. *vs. wt (10w) *P<0.05, **P<0.01. ^#^ vs. wt (4m) ^#^P<0.05, ^##^P<0.01.

The data at 10 weeks of age are derived from ref. 31.

Osteocytes have been considered to suppress bone resorption because osteocyte death is eventually followed by bone resorption [8], [21], [22]; however, apoptotic and necrotic death markers can concomitantly be present in the same cell [23]; progression to secondary necrosis could ensue in apoptotic osteocytes that are protected in the bone from phagocytosis [24], [25]; molecules that can elicit necrosis-induced immune signaling or inflammation are released from the plasma membrane during necrosis to actively recruit a defensive or reparative response in regions that have sustained damage [26]; and osteoclasts are highly regulated to respond appropriately to inflammatory changes in their microenvironment [27]. Thus, it is necessary to reconsider whether bone resorption after osteocyte death is due to osteocyte necrosis or to the function of osteocytes itself; however, the anatomic sites of osteocytes, which are embedded in bone matrix, and lack an appropriate in vitro system or animal model, have made clarification of osteocyte functions difficult. Recently, the involvement of osteocytes in osteoclastogenesis and bone resorption was reported using conditional knockout mice of β*-catenin* by *Dmp1*-Cre, which resulted in enhanced bone resorption, and conditional knockout mice of receptor activator of NF-κB ligand (*Rankl*) by *Dmp1*-Cre, which resulted in osteopetrosis [28], [29], [30].

We generated osteoblast-specific *BCL2* transgenic mice. Overexpression of *BCL2* inhibited osteoblast maturation, and the osteocytes, in which the transgene was down-regulated, gradually died by apoptosis during bone development and terminal deoxynucleotidyl transferase-mediated dUTP nick end-labeling (TUNEL)-positive lacunae accumulated in the bone [31]. As the level of transgene expression in osteoblasts was low and TUNEL-positive lacunae were most accumulated at 4 months of age, we considered that *BCL2* transgenic mice at 4 months of age might be an appropriate model for the evaluation of osteocyte functions. To pursue the functions of the osteocyte network at physiological and unloaded conditions, therefore, we investigated how destruction of the osteocyte network had influenced osteoblasts, osteoclasts, and bone mass under physiological and unloaded conditions using *BCL2* transgenic mice at 4 months of age.

**Figure 1:**
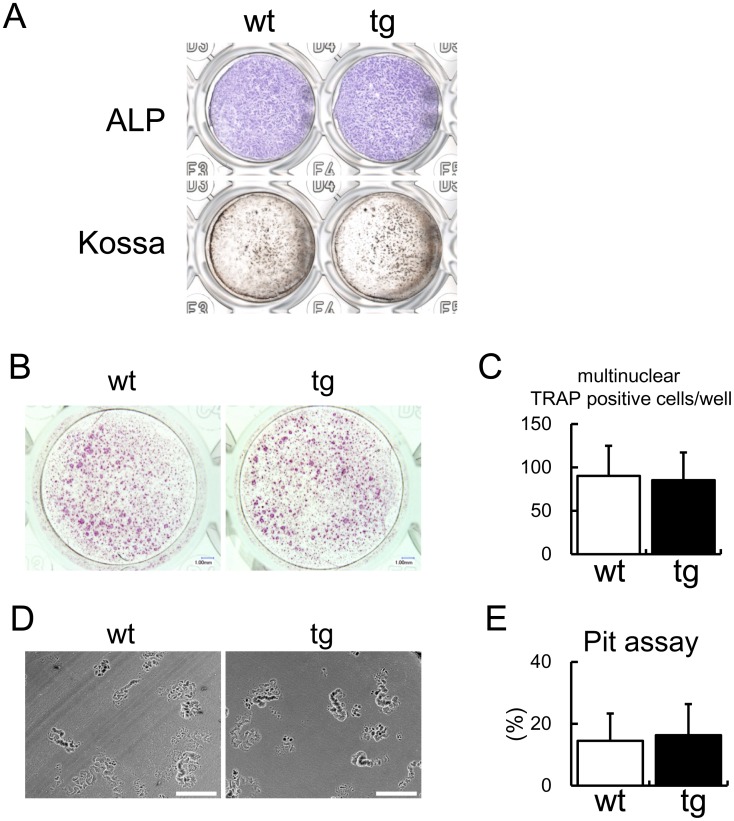
Osteoblast differentiation and osteoclastogenesis in vitro. (A) ALP activity and mineralization. Primary osteoblasts from wild-type and *BCL2* transgenic mice were seeded on 48-well plates at a density of 8×10^4^/well and ALP staining and von Kossa staining were performed after culture for 4 days and 8 days, respectively. Sixteen wild-type and 13 *BCL2* transgenic newborn mice were used in two independent experiments, and representative data are shown. (B–D) Co-culture of BMMs and primary osteoblasts. BMMs from wild-type mice were co-cultured with primary osteoblasts from wild-type or *BCL2* transgenic mice. TRAP staining was performed after 6 days (B), and the number of multinucleated TRAP-positive cells was counted (C). The resorption activity of the osteoclasts was examined by Pit assay (D), and the resorption pits were measured after 6 days (E). Scale bars = 200 µm. Data are the mean ± S.D. of 5–8 mice. Similar results were obtained in two independent experiments and representative data are shown.

## Materials and Methods

### Ethics Statement

Prior to the study, all experiments were reviewed and approved by the Animal Care and Use Committee of Nagasaki University Graduate School of Biomedical Sciences. (Permit Number: 0906170767-4).

**Figure 2:**
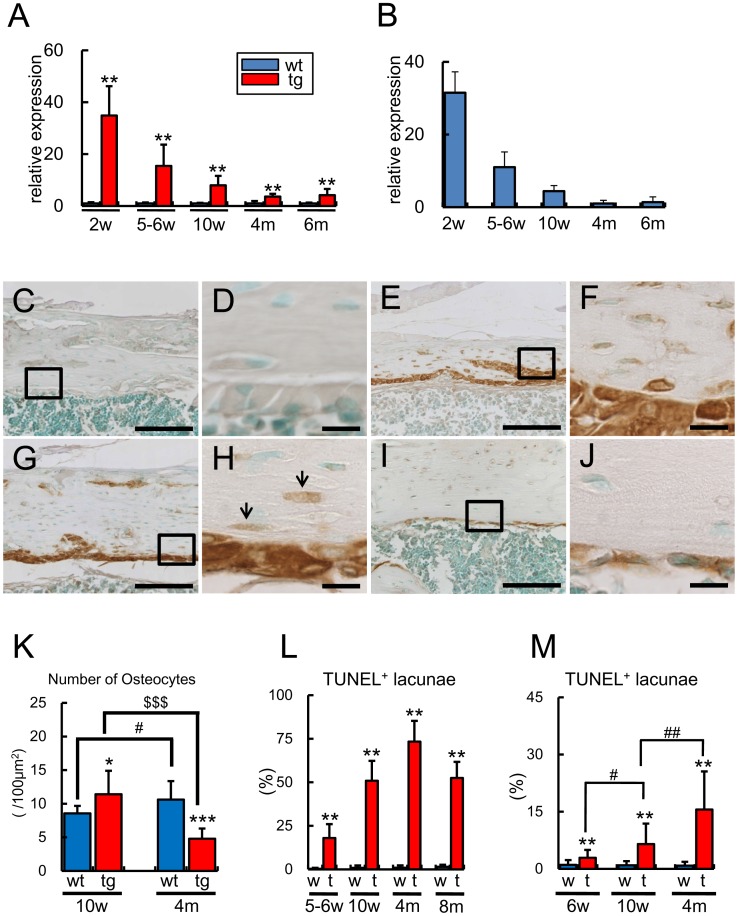
Transgene expression, osteocyte number, and the frequencies of TUNEL-positive lacunae. (A, B) Real-time RT-PCR analyses of the expression of transgene (A) and *Col1a1* (B). The expression levels of the transgene and *Col1a1* were examined using RNA that had been extracted from the whole femurs at 2 weeks of age [wt, 8 mice; tg, 7 mice] and osteoblast-enriched samples at 5–6 weeks [wt, 13 mice; tg, 11 mice], 10 weeks [wt, 8 mice; tg, 5 mice], and 4 [wt, 3 mice; tg, 9 mice] and 6 [wt, 5 mice; tg, 8 mice] months of age. The values of wild-type mice were defined as 1, and relative levels are shown in A. The value at 4 months of age was defined as 1, and relative levels are shown in B. (C–J) Immunohistochemical analysis. Sections of wild-type mice at 2 weeks of age (C) and *BCL2* transgenic mice at 2 weeks (E), 6 weeks (G), and 4 months (I) of age were reacted with anti-BCL2 antibody. Boxed regions in C, E, G, and I are magnified in D, F, H, and J, respectively. The arrows in H indicate immature osteocytes, which expressed the transgene. The lacunae, which were TUNEL-positive and contained cellular debris of dead osteocytes, were non-specifically reacted with anti-BCL2 antibody in *BCL2* transgenic mice. Scale bars  = 100 µm (C, E, G, I); 10 µm (D, F, H, J). (K) The number of osteocytes in cortical bone. The number of osteocytes was counted in the cortical bone of femurs at 10 weeks [wt, 9 mice; tg, 12 mice] and 4 months [wt, 14 mice; tg, 13 mice] of age. (L and M) Frequencies of TUNEL-positive lacunae in cortical bone (L) and trabecular bone (M). TUNEL-positive lacunae were counted in femurs at 5–6 weeks [wt, 5 mice; tg, 6 mice], 10 weeks [wt, 7 mice; tg, 9 mice], and 4 [wt, 4 mice; tg, 4 mice] and 6 [wt, 4 mice; tg, 7 mice] months of age. The number of TUNEL-positive lacunae was presented as a percentage of the total number of lacunae. In A, B, and K–M, data are presented as the mean ± S.D. *vs. wild-type mice. *P<0.05, **P<0.01, ***P<0.001, ^#^P<0.05,^ ##^P<0.01, ^$$$^P<0.001.

**Figure 3:**
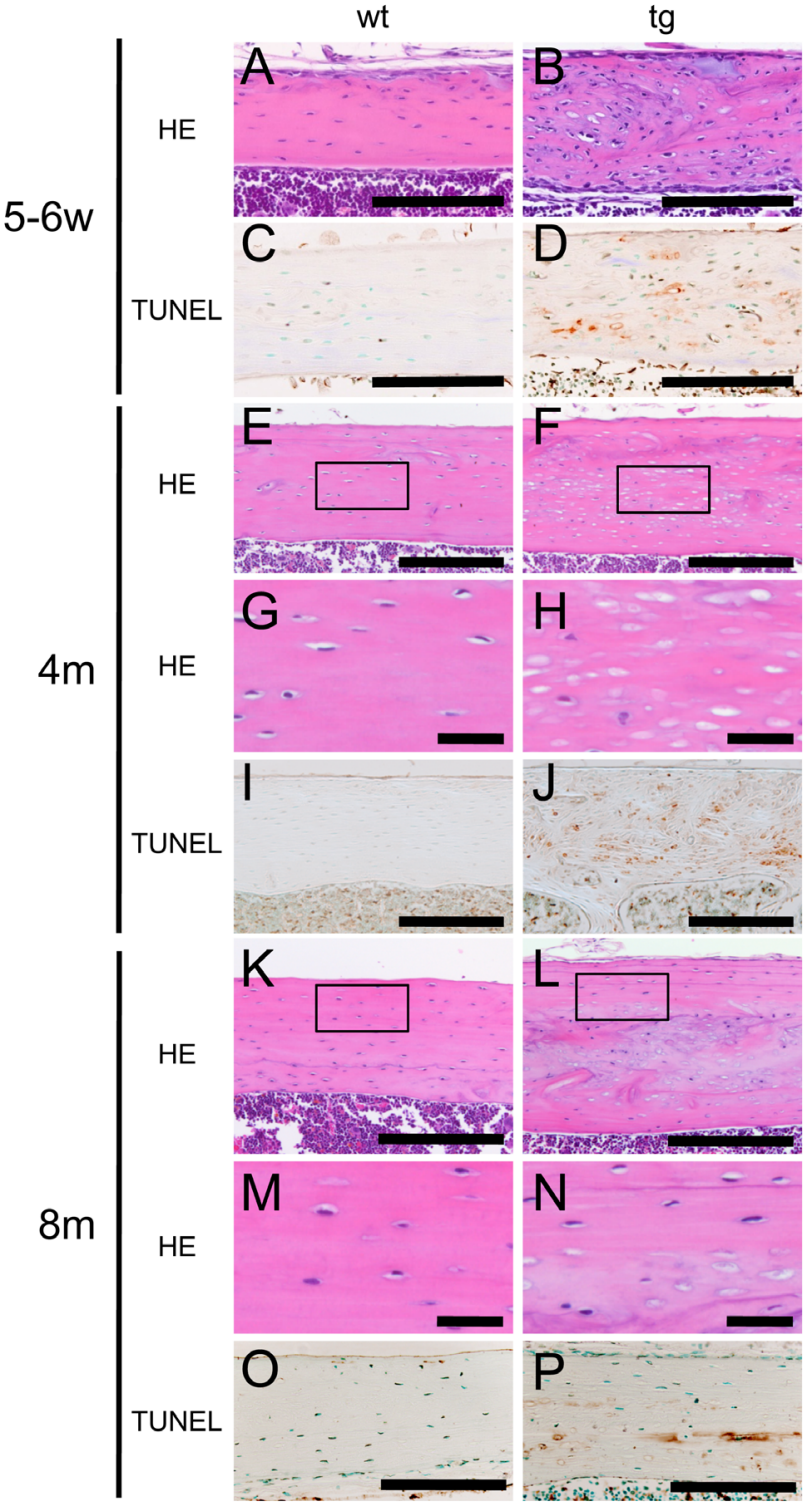
Osteocyte apoptosis in cortical bone. H–E (A, B, E–H, K–N) and TUNEL (C, D, I, J, O, P) staining of cortical bone at the diaphyses of femurs of wild-type mice (A, C, E, G, I, K, M, O) and *BCL2* transgenic mice (B, D, F, H, J, L, N, P) at 5–6 weeks (A–D), 4 months (E–J), and 8 months of age (K–P). Boxed regions in E, F, K, and L are magnified in G, H, M, and N, respectively. At 8 months of age, osteocytes with a normal appearance are located in the periphery of the cortical bone of *BCL2* transgenic mice (L, N). Scale bars  = 0.1 mm (A–F, I–L, O, P); 20 µm (G, H, M, N).

### Animal Study

Two *BCL2* transgenic mouse lines were established as previously described [31], and *BCL2* transgenic mouse line with low expression was used in this paper. The serum level of osteocalcin was examined using BTI Mouse Osteocalcin EIA kit (Biomedical Technologies Inc., Stoughton, MA), and the serum level of tartrate-resistant acid phosphatase 5b (TRAP5b) was examined using the mouse TRAP Assay (Immunodiagnostic Systems, Boldon, UK). At 6 weeks or 4 months of age, wild-type mice and tg(L) were each divided into control and unloaded groups. Unloading of the hind limbs was performed by tail suspension for 3–14 days in the unloaded group, while the mice in the control group were normally loaded, as previously described [32]. After tail suspension of the mice in the unloaded group, the mice in the control and unloaded groups were immediately anesthetized and sacrificed.

### Cell Culture Experiments

Primary osteoblasts were isolated from newborn calvaria by sequential digestion with 0.1% collagenase A and 0.2% dispase. Osteoblastic cells from the third to fifth fraction were pooled and used for osteoblast differentiation and osteoclastogenesis. To examine osteoblast differentiation, staining for alkaline phosphatase (ALP) activity and mineralization was performed as previously described [31]. The co-culture of primary osteoblasts and bone marrow-derived monocyte/macrophage lineage cells (BMMs) was performed as previously described [33].

**Figure 4:**
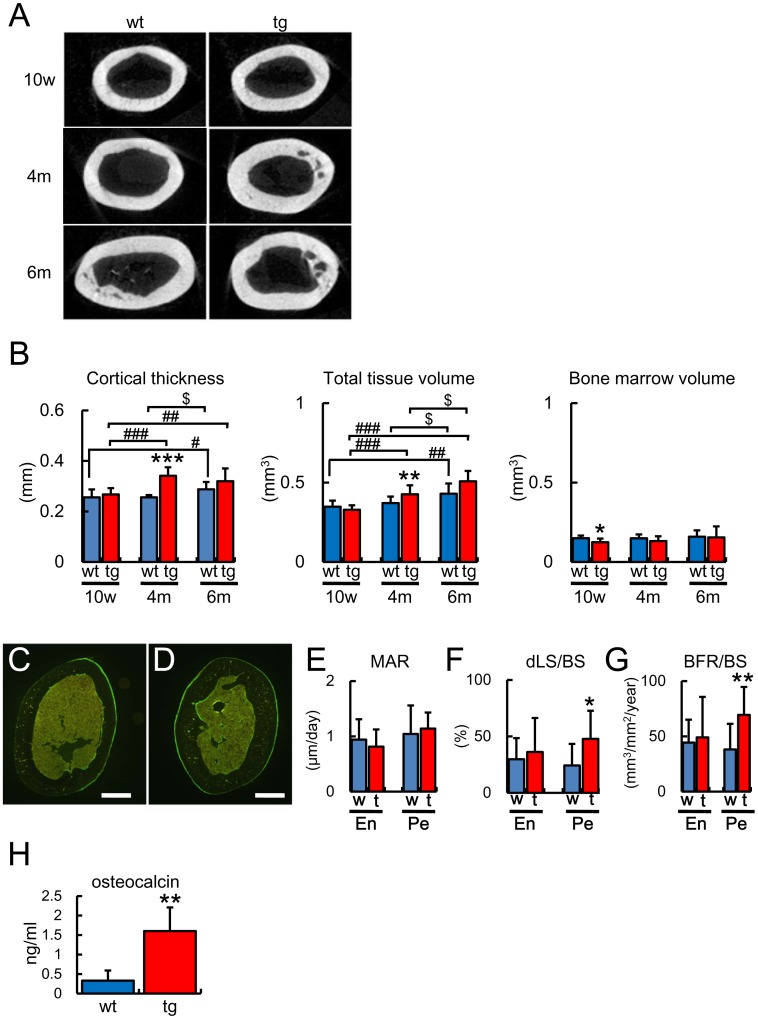
Micro-CT and bone histomorphometric analyses of cortical bone (A, B) Micro-CT analysis. Micro-CT images of mid-diaphyses of femurs (A) and cortical thickness, total tissue volume, and bone marrow volume (B) in male wild-type mice (wt) and *BCL2* transgenic mice (tg) at 10 weeks [wt, 17 mice; tg, 7 mice], 4 months [wt, 14 mice; tg, 10 mice], and 6 months [wt, 6 mice; tg, 6 mice] of age. Data are presented as the mean ± S.D. (C–G) Dynamic histomorphometric analysis of cortical bone at 4 months of age. C and D, Cross-sections from the mid-diaphyses of femurs of male wild-type mice (C) and *BCL2* transgenic mice (D), in which calcein had been injected twice. Scale bars = 0.5 mm. E–G, Mineral apposition rate (MAR) (E), double-labeled surface (dLS/BS) (F), and bone formation rate (BFR/BS) (G) in the endosteum (En) and periosteum (Pe) at the mid-diaphyses of femurs of wild-type mice (w, blue) and *BCL2* transgenic mice (t, red). Data are the mean ± S.D. of 10 mice. *vs. wild-type mice. *, ♯, $ P<0.05; **, ♯♯ P<0.01; ***, ♯♯♯ P<0.001. (H) Comparison of the serum osteocalcin level in male four wild-type mice and five *BCL2* transgenic mice at 4 months of age. Data are presented as the mean ± S.D. *vs. wild-type mice. **P<0.01.

**Figure 5:**
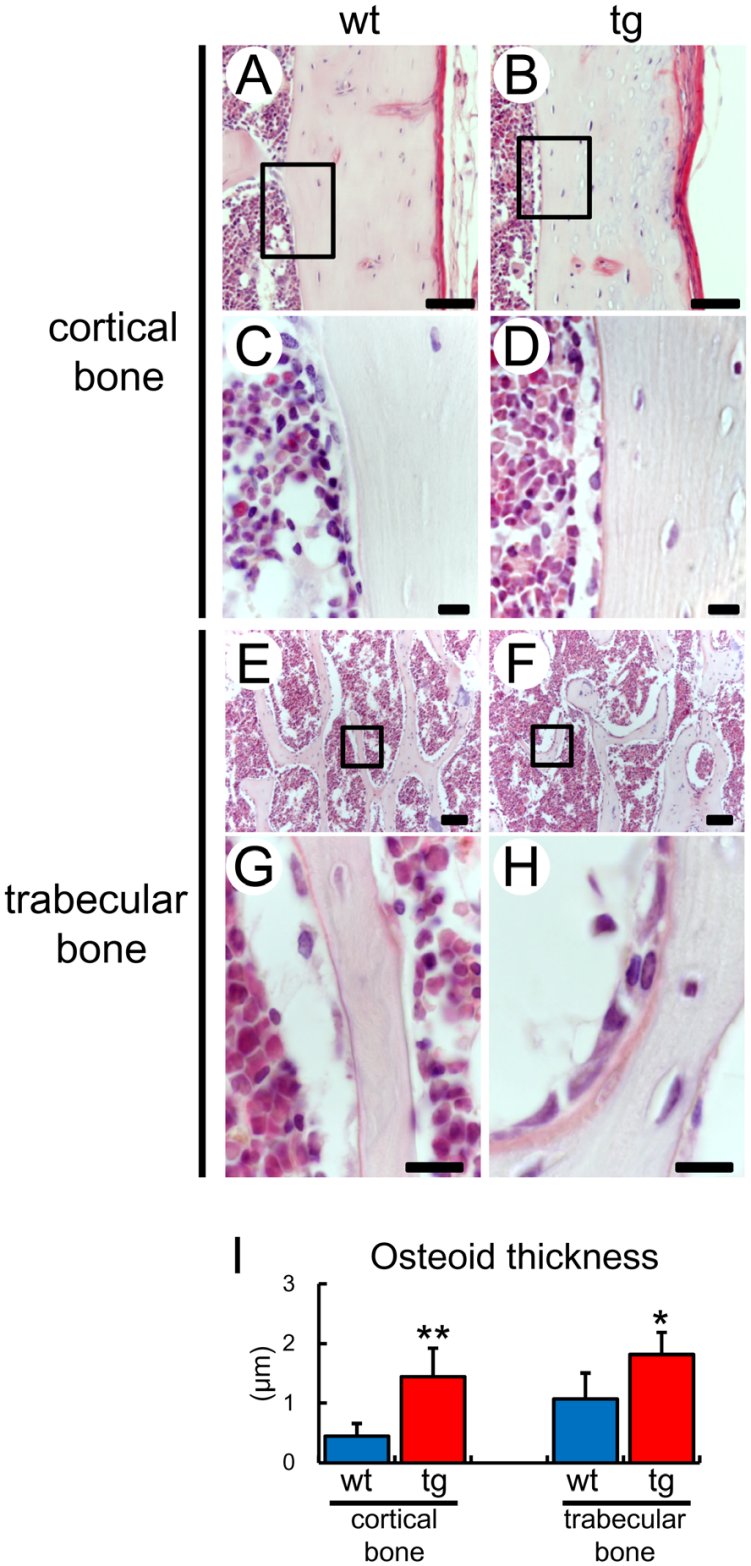
Increase of osteoid in *BCL2* transgenic mice at 4 months of age. Cortical bone (A–D) and trabecular bone (E–H) of femurs in wild-type (A, C, E, G) and *BCL2* transgenic (B, D, F, H) mice at 4 months of age. The boxed regions in A, B, E, and F are magnified in C, D, G, and H, respectively. Osteoid was visualized by Goland-Yoshilki method. Scale bars = 50 µm (A, B, E, F); 10 µm (C, D, G, H). (I) Osteoid thickness. Data are presented as the mean ± S.D. *vs. wild-type mice. *P<0.05, **P<0.01. wt, 4 mice; tg, 5 mice.

### Micro-CT Analysis

Dissected femurs at 6 weeks, 10 weeks, 4 months, or 6 months of age were analyzed by a micro-CT system (µCT-20; Scanco Medical, Brüttisellen, Switzerland). Data from scanned slices were used for three-dimensional analysis to calculate femoral morphometric parameters. Trabecular bone parameters were measured using the distal femoral metaphysis. Approximately 2.4 mm (0.5 mm from the growth plate) was cranio-caudally scanned and 200 slices were taken at 12 µm intervals. Cortical thickness was measured at the mid-diaphyses of femurs. We used a threshold value of 275 to binarize the spongiosa and cortex in wild-type and *BCL2* transgenic mice at all ages.

**Figure 6:**
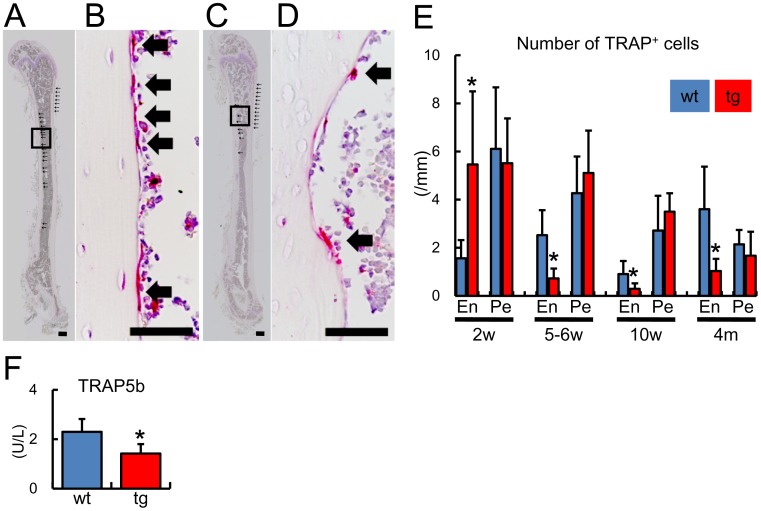
Bone resorption in *BCL2* transgenic mice. (A–E) Number of osteoclasts in cortical bone. A–D, Sections of femurs stained with TRAP in male wild-type mice (A, B) and *BCL2* transgenic mice (C, D) at 4 months of age. Boxed regions in A and C are magnified in B and D, respectively. Arrows show TRAP-positive cells. Sections were counterstained with hematoxylin. Scale bars = 0.5 mm (A, C); 50 µm (B, D). E, Number of TRAP-positive cells in the endosteum (En) and periosteum (Pe) of femurs of male wild-type mice (blue) and *BCL2* transgenic mice (red) at 2 weeks [wt, 4 mice; tg, 5 mice], 5–6 weeks [wt, 3 mice; tg, 4 mice], 10 weeks [wt, 6 mice; tg, 5 mice], and 4 months [wt, 5 mice; tg, 5 mice] of age. Data are presented as the mean ± S.D. (F) Comparison of the serum TRAP5b level in male three wild-type mice and five *BCL2* transgenic mice at 4 months of age. Data are presented as the mean ± S.D. *vs. wild-type mice. *P<0.05.

### Histological Analysis

For histological analyses of the long bones, mice were sacrificed and fixed in 4% paraformaldehyde/0.01M phosphate-buffered saline (PBS), and the long bones were decalcified in 10% EDTA (pH7.4) and embedded in paraffin. For Goland-Yoshiki method to detect osteoid, the long bones were refixed with Cyanuric Chloride before decalcification. Sections (3–7 µm thick) were stained with hematoxylin and eosin (H–E), stained for TRAP activity, or subjected to immunohistochemistry using monoclonal anti-human BCL2 antibody (Abcam, Cambridge, UK) or anti-Sost antibody (R&D, Minneapolis, MN). TUNEL staining was performed using the ApopTag® Peroxidase In Situ Apoptosis Detection Kit S7100 (Chemicon, Billerica, MA) according to the manufacturer’s instructions. For assessment of dynamic histomorphometric indices, mice were injected with calcein at 10 d and 2 d before sacrifice at a dose of 0.16 mg/10 g body weight. Bone histomorphometric analyses were performed using femurs as previously described [34].

**Figure 7:**
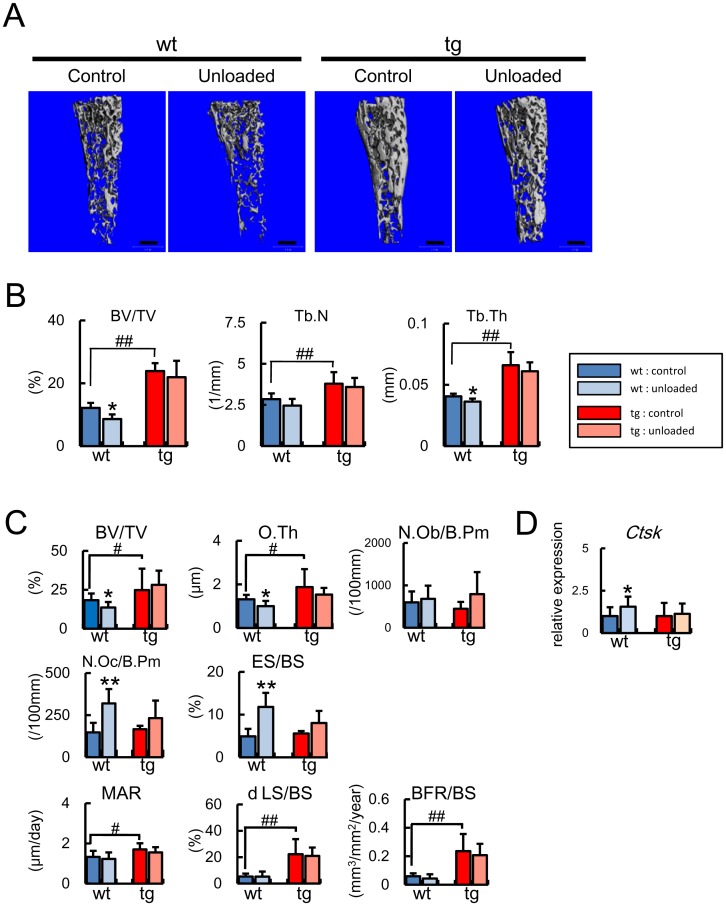
Micro-CT, bone histomorphometry, and real-time RT-PCR analyses after unloading at 4 months of age. (A, B) Micro-CT analysis. Tail suspension was performed for 2 weeks using male wild-type mice [control group, 13 mice; unloaded group, 9 mice] and *BCL2* transgenic mice [control group, 8 mice; unloaded group, 8 mice] at 4 months of age. A, Micro-CT images of femurs. Scale bars = 0.5 mm. B, Trabecular bone volume (BV/TV), trabecular number (Tb.N), and trabecular thickness (Tb.Th) were evaluated by micro-CT. (C) Bone histomorphometrical analysis of trabecular bone. The trabecular bone volume (BV/TV), osteoid thickness (O.Th), number of osteoblasts (N.Ob/B.Pm), number of osteoclasts (N.Oc/B.Pm), eroded surface (ES/BS), mineral apposition rate (MAR), double-labeled surface (dLS/BS), and bone formation rate (BFR/BS) were measured on distal femoral metaphysis in wild-type mice [control group, 8 mice; unloaded group, 11 mice] and *BCL2* transgenic mice [control group, 8 mice; unloaded group, 6 mice] at 4 months of age. (D) *Ctsk* expression. Tail suspension was performed for 3 days and *Ctsk* expression was examined by real-time RT-PCR analysis using osteoblast-enriched samples from wild-type mice [control group, 9 mice; unloaded group, 11 mice] and *BCL2* transgenic mice [control group, 6 mice; unloaded group, 5 mice] at 4 months of age. The values of the control groups were defined as 1, and relative levels are shown. In B–D, data are presented as the mean ± S.D. *vs. control. *, ♯ P<0.05; **, ♯♯ P<0.01.

### Real-time RT-PCR and Western Blot Analyses

Muscle, connective tissue, and periosteum were removed from femurs and tibiae, and the bones were cut at the metaphyses. After hematopoietic cells in the diaphyses of femurs and tibiae were flushed out with PBS, osteoblast-enriched cells were collected using a micro-intertooth brush (Kobayashi Pharmaceutical Co. Ltd., Osaka, Japan). The remaining bone was used as a source of osteocyte-enriched cells. At the beginning of each experiment, nearly complete removal of osteoblasts from the endosteum by the micro-intertooth brush was confirmed using a scanning electron microscope (Miniscope TM-1000; Hitachi) (Fig. S1). Total RNA was extracted using ISOGEN (Wako, Osaka, Japan), and real-time RT-PCR was performed using the following primers as previously described [35]. Mouse *Bcl2 and human BCL2*, 5′-GAGGATTGTGGCCTTCTTTG-3′ and 5′-CGTTATCCTGGATCCAGGTG-3′; human *BCL2* 5′-CCGCGACTTCGCCGAGATGT-3′ and 5′-GGTTGACGCTCTCCACACAC-3′; *Col1a1*, 5′-CCTGGAATGAAGGGACACCG-3′ and 5′-CCATCGTTACCGCGAGCACC-3′; *Ctsk*, 5′-CAGCAGAGGTGTGTACTATG-3′ and 5′-GCGTTGTTCTTATTCCGAGC-3′; *Dmp1*, 5′-GGCTGTCCTGTGCTCTCCCAG-3′ and 5′-GGTCACTATTTGCCTGTGCCTC-3′; *Sost*, 5′-CTTCAGGAATGATGCCACAGAGGT-3′ and 5′-ATCTTTGGCGTCATAGGGATGGTG-3′; *Fgf23*, 5′-ACTTGTCGCAGAAGCATC-3′ and 5′-GTGGGCGAACAGTGTAGAA-3′; *Mepe*, 5′-CAGTGGCTCCCCAGATCTTC-3′ and 5′-GCTTTCAGGACCAGACCCAG-3′; *Phex*, 5′-GTGCATCTACCAACCAGATACG-3′ and 5′-TCTGTTCCCCAAAAGAAAGG-3′; *keratocan*, 5′-TCCCCCATCAACTTATTTTAGC-3′ and 5′-GGTTGCCATTACAGCACCTT-3′; *Runx2*, 5′-GAGAGGTACCAGATGGGACT-3′ and 5′-CACTTGGGGAGGATTTGTGA-3′; *Osterix*, 5′-AGGCACAAAGAAGCCATAC-3′ and 5′-AATGAGTGAGGGAAGGGT-3′; *osteocalcin*, 5′-CGCTCTGTCTCTCTGACCTC-3′ and 5′-GACTGAGGCTCCAAGGTAGC-3′; *Rankl*, 5′-CAAGCTCCGAGCTGGTGAAG-3′ and 5′-CCTGAACTTTGAAAGCCCCA-3′; *Opg*, 5′-AAGAGCAAACCTTCCAGCTGC-3′ and 5′-CACGCTGCTTTCACAGAGGTC-3′; *Dkk1*, 5′-AGTGTGGCGCCGGGAGTTCT-3′ and 5′-TACACCTCCGACGCCGGCTG-3′; *sFRP1*, 5′-TGCGAGCCGGTCATGCAGTT-3′ and 5′-ACTCGTTGTCGCATGGAGGA-3′; *sFRP2*, 5′-GGACGACAACGACATCATGG-3′ and 5′-CAGGCTTCACACACCTTGGG-3′; *sFRP4*, 5′-GCCGTCCAGAGGAGTGGTTG-3′ and 5′-TGGGGCAGGATATGTGGACA-3′; *sFRP5*, 5′-CTGATGGCCTCATGGAACAG-3′ and 5′-GCTTTAAGGGGCCTGCCTTG-3′; *Gapdh*, 5′-TGCACCACCAACTGCTTAG-3′ and 5′-GAACATCATCCCTGCATCC-3′. We normalized the values to that of *Gapdh*. Western blot analysis was performed using anti-β-catenin (BD Biosciences, San Jose, CA) and anti-β-actin (Santa Cruz Biotechnology, Santa Cruz, CA) antibodies.

### Statistical Analysis

Statistical analyses were performed by Student’s *t*-test using Ekuseru-Toukei 2010 (Social Survey Research Information Co., Ltd., Tokyo, Japan). P<0.05 was considered significant.

## Results

### The Effects of *BCL2* Transgene in Osteoblast Differentiation and Osteoclastogenesis

We established two lines of human *BCL2* transgenic mice under the control of mouse 2.3 kb *Col1a1* promoter with different expression levels, and both lines showed osteocyte apoptosis probably due to the reduced osteocyte processes [31]. The transgenic line with low expression of *BCL2* was used to analyze the effect of osteocyte death, because the functions of osteoblasts in these mice were less affected by the transgene than those in the transgenic line with high *BCL2* expression [31]. The bone formation rate and osteoid thickness were reduced in the transgenic line with high *BCL2* expression but not the transgenic line with low *BCL2* expression at 10 weeks of age (Figure S2) [31]. However, the function of osteoblasts in the transgenic line with low *BCL2* expression was mildly impaired at 10 weeks of age, because the bone volume and bone formation rate were similar to those in wild-type mice irrespective of the increased osteoblast density, while the osteoclastogenesis was normal (Table 1) [31]. In osteoblast differentiation in vitro, primary osteoblasts from the transgenic line with low *BCL2* expression showed similar levels of ALP activity and mineralization compared with those from wild-type mice (Fig. 1A), although primary osteoblasts from the transgenic line with high *BCL2* expression showed lower ALP activity and mineralization compared with those from wild-type mice [31]. We also examined osteoclast differentiation by co-culturing primary osteoblasts and BMMs. The number of TRAP-positive cells and the resorption area were similar between the co-culture of primary osteoblasts from the transgenic line with low *BCL2* expression and wild-type BMMs and the co-culture of wild-type primary osteoblasts and BMMs (Fig. 1B–E). Further, we previously showed that retroviral introduction of *BCL2* into wild-type primary osteoblasts had no effect on osteoclastogenesis in the co-culture with wild-type bone marrow cells [31].

**Figure 8:**
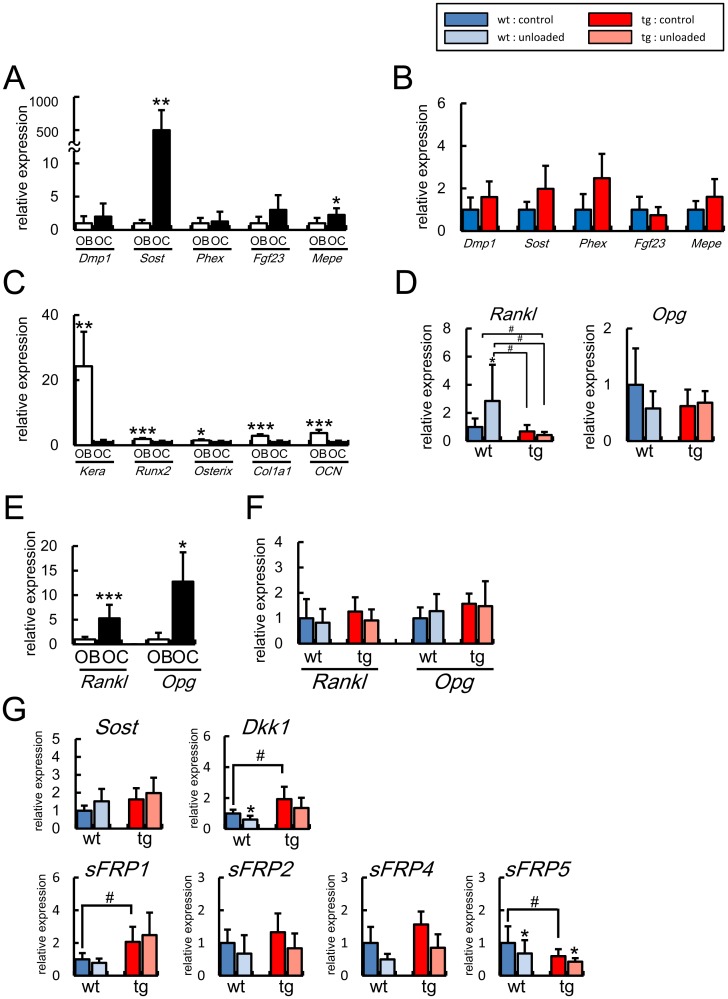
Real-time RT-PCR analyses of the expression of osteocyte and osteoblast marker genes, *Rankl*, *Opg*, and Wnt antagonist genes at 4 months of age. (A) Expression of *Dmp1*, *Sost*, *Phex*, *Fgf23*, and *Mepe* in the osteoblast fractions (OB, n = 6) and osteocyte fractions (OC, n = 9) of wild-type mice. The values of osteoblast fractions were defined as 1, and relative levels are shown. *vs. osteoblast fractions. (B) Expression of *Dmp1*, *Sost*, *Phex*, *Fgf23*, and *Mepe* in control groups of wild-type mice (n = 4) and *BCL2* transgenic mice (n = 5). The values of wild-type mice were defined as 1, and relative levels are shown. (C) Expression of osteoblast marker genes. Expressions of *keratocan* (*Kera*), *Runx2*, *Osterix*, *Col1a1*, and *osteocalcin* (*OCN*) were examined by real-time RT-PCR using osteoblast-enriched samples and osteocyte-enriched samples from 7 wild-type mice at 6 weeks of age. The values of osteocyte fractions were defined as 1, and relative levels are shown. *vs. osteocyte fractions. (D) *Rankl* and *Opg* expression in osteoblast-enriched samples after unloading. Tail suspension was performed for 3 days using male wild-type mice [control group, 8 mice; unloaded group, 9 mice] and *BCL2* transgenic mice [control group, 12 mice; unloaded group, 6 mice] at 4 months of age. (E) Expression of *Rankl* and *Opg* in the osteoblast fractions (OB) and osteocyte fractions (OC) from 6 and 11 wild-type mice at 4 months of age, respectively. The values of osteoblast fractions were defined as 1, and relative levels are shown. *vs. osteoblast fractions. (F) Expression of *Rankl* and *Opg* in osteocyte-enriched samples. Tail suspension was performed for 3 days using male wild-type mice [control group, 9 mice; unloaded group, 8 mice] and *BCL2* transgenic mice [control group, 12 mice; unloaded group, 11 mice] at 4 months of age. (G) Expression of Wnt antagonist genes in osteocyte-enriched samples after unloading. Tail suspension was performed for 3 days using male wild-type mice and *BCL2* transgenic mice at 4 months of age (8 mice in each group). The values of the control group of wild-type mice were defined as 1, and relative levels are shown in D, F, and G. In A–G, data are presented as the mean ± S.D. *vs. control in D, F, and G. *, ♯ P<0.05; **P<0.01; ***P<0.001.

### Osteocyte Apoptosis in the Transgenic Line with Low *BCL2* Expression

The transgene expression was dependent on age; it was high in mice at 2 weeks of age, but it gradually fell during growth and was low in mice at 4–6 months of age (Fig. 2A). The change in the expression level of the transgene during aging was similar to those of *Col1a1* in wild-type mice (Fig. 2B). In immunohistochemical analysis using anti-human BCL2 antibody, which reacts on human BCL2 but not mouse Bcl2, the transgene expression was strongly detected in osteoblasts and mildly in most of osteocytes at 2 weeks of age (Fig. 2C–F). At 6 weeks of age, the transgene expression was also strongly detected in osteoblasts, but its expression in osteocytes was restricted to immature osteocytes, which were located near the surface of cortical bone (Fig. 2G, H). At 4 months of age, the transgene expression was still clearly detected in osteoblasts but undetectable in osteocytes (Fig. 2I, J). The numbers of osteocytes were increased in *BCL2* transgenic mice compared with wild-type mice in both cortical and trabecular bone until 10 weeks of age probably due to the increase of osteoblast density, whereas they were reduced in *BCL2* transgenic mice compared with wild-type mice in both cortical and trabecular bone at 4 months of age (Figs. 2K, 3B) (Table 1) [31]. Unexpectedly, TUNEL-positive lacunae accumulated during aging. About 1% of lacunae in the cortical bone at the diaphyses of femurs were TUNEL-positive in wild-type mice from 5 weeks to 8 months of age, whereas the corresponding percentage in *BCL2* transgenic mice was about 20% at 5–6 weeks of age, about 50% at 10 weeks of age, about 75% at 4 months of age, and about 50% at 8 months of age (Figs. 2L and 3). After the death of osteocytes, the lacunae contained only cellular debris but TUNEL reactivity was retained in the lacunae (Fig. 3), because the debris of dead osteocytes cannot be eliminated until the surrounding bone is resorbed [22], [36]. At 6–8 months of age, the cortical bone in *BCL2* transgenic mice was partly remodeled and the remodeled bone contained osteocytes with a normal appearance, probably due to the reduction in transgene expression (Fig. 3K–P).

Bone canalicular staining showed that the severity in the disturbance of osteocyte network was different depending on the age in *BCL2* transgenic mice. Osteocyte network was disturbed at the center but not at the periphery of cortical bone at 10 weeks of age, it was disturbed in the whole area of cortical bone at 4 months of age, and the disturbance was restricted to the inner half of cortical bone at 8 months of age (Figures S3, S4, S5). The reduction in the number of canaliculi was observed in trabecular bone as well as cortical bone of *BCL2* transgenic mice until 4 months of age (Figures S3, S4, S5, S6) [31].

We also examined the frequency of TUNEL-positive lacunae in trabecular bone. About 1% of lacunae in the trabecular bone of femurs were TUNEL-positive in wild-type mice from 6 weeks to 4 months of age, whereas the corresponding percentage in *BCL2* transgenic mice was 3% at 6 weeks of age, 7% at 10 weeks of age, and 16% at 4 months of age (Fig. 2M).

These findings showed that the transgene expression was low in osteoblasts and barely detectable in osteocytes, the number of osteocytes was reduced, the frequency of TUNEL-positive lacunae peaked, and osteocyte network was most severely disturbed at 4 months of age in *BCL2* transgenic mice. Thus, we focused on *BCL2* transgenic mice at 4 months of age to evaluate bone phenotypes after the accumulation of TUNEL-positive lacunae, while minimizing the effects of BCL2 on osteoblasts and osteocytes.

### Increase in Bone Formation and Reduced Number of Osteoclasts in Cortical Bone of *BCL2* Transgenic Mice after the Accumulation of TUNEL-positive Lacunae

At 10 weeks of age, cortical thickness and total tissue volume at the mid-diaphyses of femurs in *BCL2* transgenic mice were similar to those in wild-type mice, but bone marrow volume in *BCL2* transgenic mice was less than that in wild-type mice. At 4 months of age, however, the cortical thickness and total tissue volume but not bone marrow volume had increased in *BCL2* transgenic mice but not in wild-type mice compared with at 10 weeks of age, indicating that cortical bone was enlarged due to the acquisition of bone in the periosteum (Fig. 4A, B). On dynamic bone histomorphometric analysis, the double-labeled surface and bone formation rate were increased in the periosteum but not in the endosteum of the cortical bone of femurs of *BCL2* transgenic mice compared with the respective parameter in wild-type mice at 4 months of age (Fig. 4C–G), indicating that the osteoblast function was enhanced in the periosteum. Further, the serum level of osteocalcin, which is a serum marker of bone formation, was increased in *BCL2* transgenic mice compared with wild-type mice at 4 months of age (Fig. 4H). In histological analysis, osteoid was thick in cortical bone of *BCL2* transgenic mice compared with wild-type mice at 4 months of age (Fig. 5A–D, I). However, the cortical thickness was not significantly different between wild-type and *BCL2* transgenic mice at 6 months of age (Fig. 4A, B). These findings suggest that cortical thickness increased in parallel with the reduction in the numbers of osteocytes and the accumulation of TUNEL-positive lacunae.

**Figure 9:**
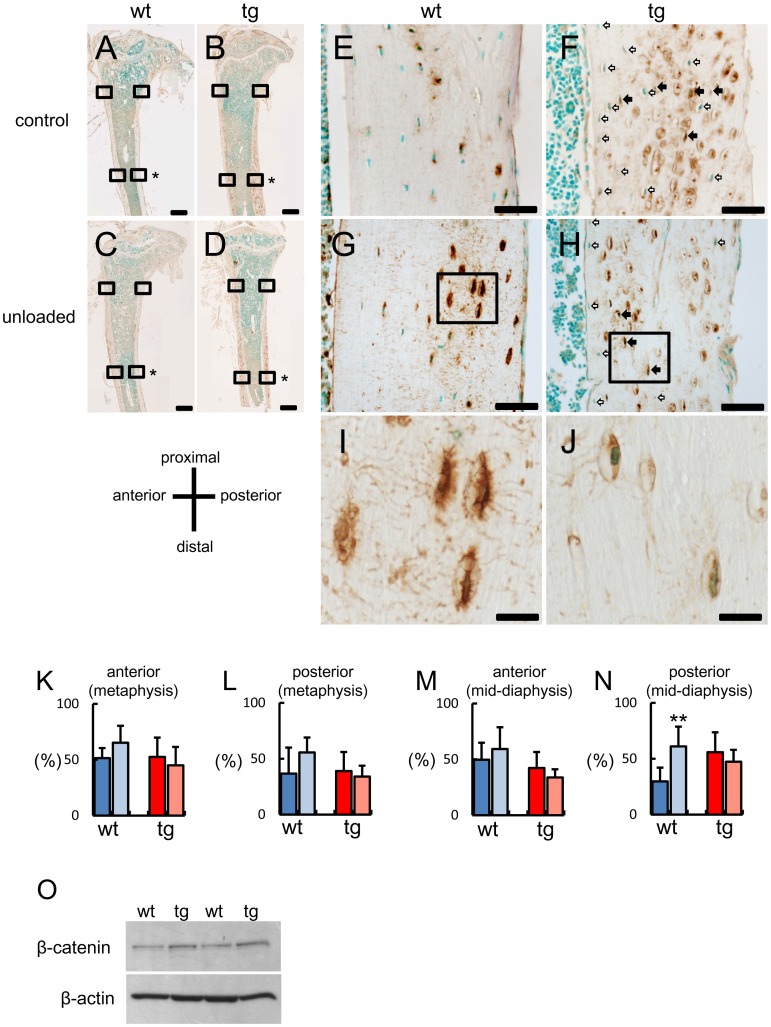
Immunohistochemical analysis of Sost after unloading. Immunohistochemistry using anti-Sost antibody in tibial sections of control (A, B, E, F) and unloaded (C, D, G–J) groups in wild-type (A, C, E, G, I) and *BCL2* transgenic (B, D, F, H, J) mice at 4 months of age. The boxed regions with asterisks in A–D are magnified in E–G, respectively. The boxed regions in G and H are magnified in I and J, respectively. In F and H, closed arrows indicate Sost-positive osteocytes and open arrows indicate Sost-negative osteocytes. The lacunae with cellular debris in *BCL2* transgenic mice were non-specifically stained with Sost antibody (F, H). The sections were counterstained with methylgreen. Note that Sost is distributed through canaliculi throughout bone in wild-type mice but not in *BCL2* transgenic mice (I, J). Scale bars = 0.5 mm (A–D); 50 µm (E–H); 10 µm (I, J). (K–N) Frequency of Sost-positive cells in cortical bone. Sost-positive cells were counted in the anterior (K, M) and posterior (L, N) sides of cortical bone at the metaphysis (K, L) and mid-diaphysis (M, N) of tibiae. Tail suspension was performed for 14 days using male wild-type mice [control group, 7 mice; unloaded group, 9 mice] and *BCL2* transgenic mice [control group, 9 mice; unloaded group, 8 mice] at 4 months of age. The number of Sost-positive osteocytes was presented as a percentage of the total number of osteocytes. Only the cells with a nucleus were counted. Data are presented as the mean ± S.D. *vs. control. **P<0.01. (O) Western blot analysis using anti-β-catenin antibody. Proteins were extracted from osteoblast fractions from wild-type and *BCL2* transgenic mice at 4 months of age. β-actin was used as an internal control.

Next, we examined bone resorption in *BCL2* transgenic mice by counting multinucleated TRAP-positive cells at 2, 5–6, and 10 weeks and 4 months of age, and by measuring the serum level of TRAP5b, which is a serum marker of bone resorption, at 4 months of age (Fig. 6). The number of osteoclasts in the periosteum of femurs was similar between wild-type mice and *BCL2* transgenic mice at all ages examined. In the endosteum, the number of osteoclasts in *BCL2* transgenic mice was increased compared with in wild-type mice at 2 weeks of age, whereas the number of osteoclasts in the endosteum in *BCL2* transgenic mice was lower than in wild-type mice at 5–6 weeks, 10 weeks and 4 months of age (Fig. 6A–E). In accordance with these findings, bone marrow volume was reduced in *BCL2* transgenic mice compared with wild-type mice at 10 weeks of age (Fig. 4B). Further, the serum level of TRAP5b was lower in *BCL2* transgenic mice than in wild-type mice at 4 months of age (Fig. 6F). These findings suggest that the reduction in osteoclastogenesis and bone resorption occurred in cortical bone in parallel with the reduction in the number of osteocytes and the accumulation of TUNEL-positive lacunae. Serum levels of calcium and phosphate were similar between wild-type mice and *BCL2* transgenic mice at 4 months of age (calcium: wild-type mice 8.16±0.24 mg/dl, *BCL2* transgenic mice 8.39±0.54 mg/dl; phosphate: wild-type mice 9.7±1.39 mg/dl, *BCL2* transgenic mice 10.8±0.97 mg/dl, n = 4–5).

### Trabecular Bone of *BCL2* Transgenic Mice was Increased Due to Enhanced Bone Formation through Augmented Osteoblast Function at 4 Months of Age

On micro-CT analysis, trabecular bone volume, trabecular number, and trabecular thickness were increased in *BCL2* transgenic mice (control group) compared with wild-type mice (control group) at 4 months of age (Figs. 7A, B), although these parameters in *BCL2* transgenic mice were similar to those in wild-type mice at 10 weeks of age [31]. Bone histomorphometric analysis of trabecular bone showed that the increase in bone volume was due to enhanced bone formation through augmented osteoblast function, because osteoblast number, osteoclast number, and eroded surface were similar between wild-type mice and *BCL2* transgenic mice, but the parameters for bone formation, including osteoid thickness, mineral apposition rate, double-labeled surface, and bone formation rate, were increased in *BCL2* transgenic mice compared with wild-type mice, indicating that the trabecular bone of *BCL2* transgenic mice was increased due to enhanced bone formation through augmented osteoblast function at 4 months of age (Table 1). Osteoid was apparently increased in trabecular bone of *BCL2* transgenic mice compared with wild-type mice at 4 months of age (Fig. 5E–I). However, osteoblast function was mildly impaired in *BCL2* transgenic mice at 10 weeks of age as shown by the increased osteoblast density but normal level of bone formation (Table 1) [31].

### No Reduction in Bone Mass at the Unloaded Condition in *BCL2* Transgenic Mice at 4 Months of Age

To further investigate the effect of the accumulation of TUNEL-positive lacunae, we performed tail suspension to generate an unloaded condition in the hind limbs using *BCL2* transgenic mice at 4 months of age and analyzed the femurs by micro-CT (Figs. 7A, B). Bone volume and trabecular thickness were reduced in the femurs of wild-type mice after unloading, whereas these parameters were unchanged in the femurs of *BCL2* transgenic mice after unloading (Fig. 7B). Bone histomorphometric analysis showed that the reduction in bone volume after unloading in wild-type mice at 4 months of age was mainly due to enhanced osteoclastogenesis, as indicated by increases in the osteoclast number and eroded surface, and partly due to reduced osteoblast function, as indicated by the decrease in osteoid thickness (Fig. 7C). In contrast, the parameters of both bone formation and bone resorption were unchanged in *BCL2* transgenic mice at 4 months of age after unloading. In accordance with the bone histomorphometric data, the expression of *Ctsk* was increased in the unloaded group compared with the control group in wild-type mice but not in *BCL2* transgenic mice, indicating that osteoclastogenesis was enhanced at unloading in wild-type mice but not in *BCL2* transgenic mice (Fig. 7D).

### Upregulation of *Rankl* Expression in Osteoblasts after Unloading in Wild-type Mice but not in *BCL2* Transgenic Mice

**Figure 10:**
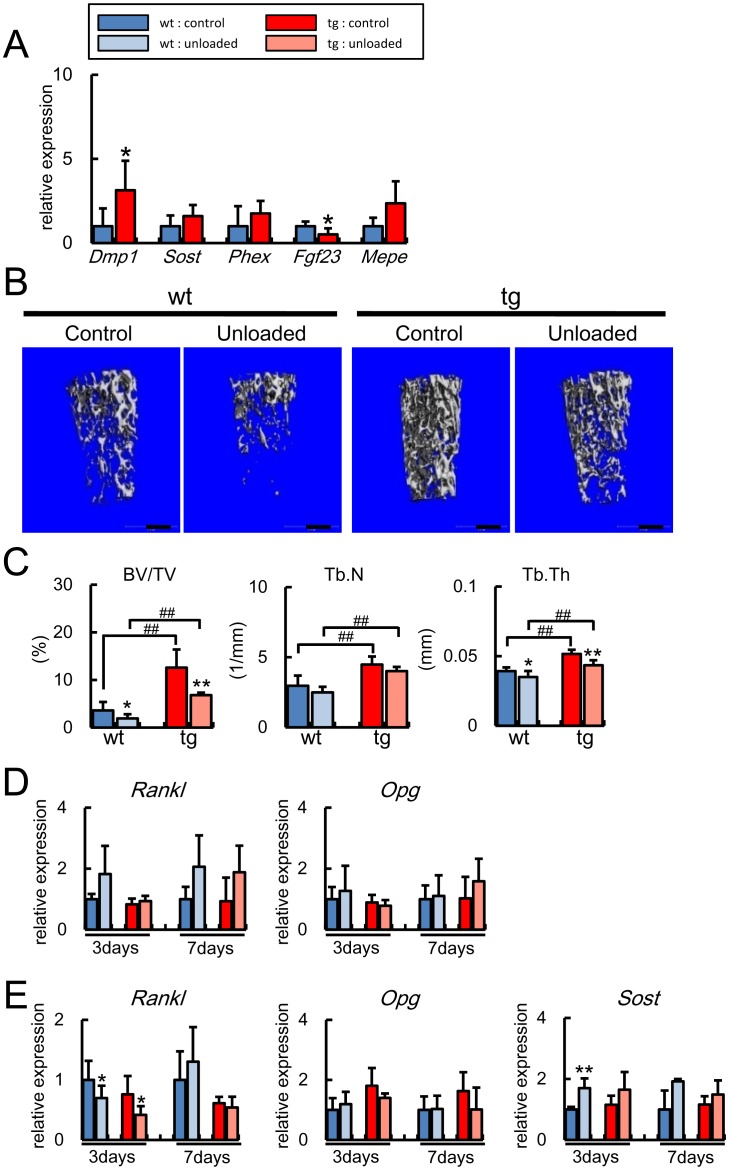
Real-time RT-PCR and micro-CT analyses at 6 weeks of age. (A) Real-time RT-PCR analysis of osteocyte marker genes. RNA was extracted from osteocyte-enriched samples of control groups of wild-type mice and *BCL2* transgenic mice at 6 weeks of age. The values of wild-type mice were defined as 1, and relative levels are shown. Data are presented as the mean ± S.D. of 3 mice. *vs. control. *P<0.05. (B, C) Micro-CT analysis of femurs. Tail suspension was performed for 1 week using male wild-type mice [control group, 10 mice; unloaded group, 8 mice] and *BCL2* transgenic mice [control group, 9 mice; unloaded group, 6 mice] at 6 weeks of age. B, Micro-CT images. Scale bars  = 0.5 mm. C, Trabecular bone volume (BV/TV), trabecular number (Tb.N), and trabecular thickness (Tb.Th) were evaluated by micro-CT. Data are presented as the mean ± S.D. *vs. control. *,♯P<0.05, **,♯♯P<0.01. (D, E) Real-time RT-PCR analysis after unloading. Tail suspension was performed for 3 days or 7 days using male wild-type mice [control group, 4 mice; unloaded group, 5 mice] and *BCL2* transgenic mice [control group, 4 mice; unloaded group, 4 mice] at 6 weeks of age, and RNA was extracted from osteoblast-enriched samples (D) and osteocyte-enriched samples (E) of the tibiae and femurs. The values of the control group of wild-type mice were defined as 1, and relative levels are shown. Data are presented as the mean ± S.D. *vs. control. *P<0.05, **P<0.01.

We prepared osteoblast-enriched samples and osteocyte-enriched samples as described in the Materials and methods. We first compared the expression of *Dmp1*, *Sost*, *Phex*, *Fgf23*, and *Mepe,* which are highly expressed in osteocytes [37], between the osteoblast-enriched samples and osteocyte-enriched samples prepared from wild-type mice at 4 months of age (Fig. 8A). *Sost* expression was specifically detected in the osteocyte fractions, *Mepe* expression was significantly high in the osteocyte fractions, and the levels of *Dmp1* and *Fgf23* expression were marginally high in the osteocyte fractions compared with the osteoblast fractions, while *Phex* expression was similar in the osteoblast and osteocyte fractions. Although we previously reported that Dmp1 is expressed in osteocytes [38], *Dmp1* expression was also detected in the osteoblast fractions, because Dmp1 was detected in osteoblasts, which are going to be embedded in the bone matrix, as well as osteocytes by immunohistochemistry (unpublished data). The expressions of these genes in individual osteocyte, which remained to be alive in *BCL2* transgenic mice, were examined using the osteocyte fractions. These expressions in the osteocyte fractions of *BCL2* transgenic mice were comparable to those in wild-type mice at 4 months of age (Fig. 8B). We also compared the expression of the genes, which were dominantly expressed in osteoblasts, between osteoblast-enriched samples and osteocyte-enriched samples to examine their purity. *Keratocan* was specifically detected in osteoblast-enriched samples as previously reported [39]. The expressions of *Runx2*, *Osterix*, *Col1a1*, and *osteocalcin* were also significantly higher in osteoblast-enriched samples than osteocyte enriched samples (Fig. 8C).

**Figure 11:**
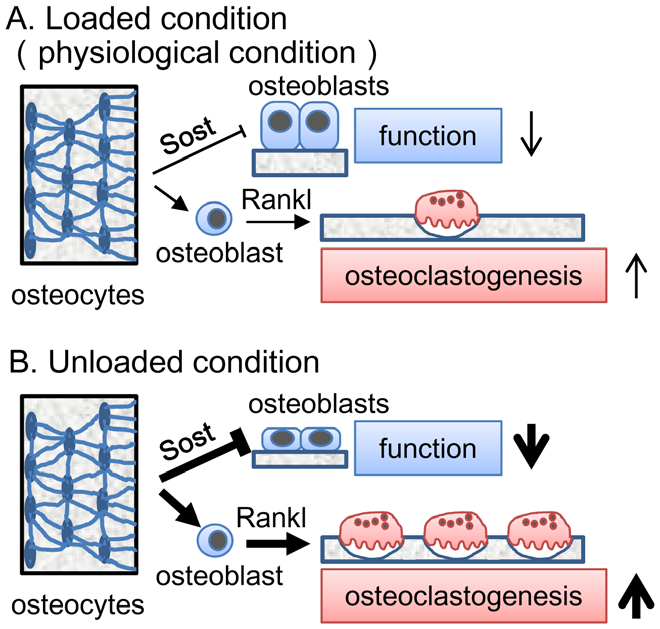
A model of osteocyte functions. (A) In the loaded (physiological) condition, the osteocyte network inhibits osteoblast function, enhances osteoclastogenesis, and negatively regulates bone mass. (B) In the unloaded condition, the effect of osteocyte network on osteoblast function is augmented through the induction of Sost in osteocytes and that on osteoclastogenesis is augmented through the induction of Rankl in osteoblasts, resulting in reduced bone mass. The thickness of the lines and arrows in A and B reflects the strength of the effects.

After unloading, *Rankl* expression in osteoblasts was upregulated in wild-type mice but not in *BCL2* transgenic mice at 4 months of age (Fig. 8D). Further, the *Rankl* expression was significantly lower in *BCL2* transgenic mice in the unloaded condition compared with that in wild-type mice in the control condition (Fig. 8D). The expression of *Osteoprotegerin (Opg)* in osteoblasts was not affected by unloading in either wild-type mice or *BCL2* transgenic mice at 4 months of age (Fig. 8D). As Rankl is expressed in osteocytes as well as osteoblasts [40], we compared the expression levels of *Rankl* and *Opg* between osteoblast and osteocyte fractions from wild-type mice at 4 months of age (Fig. 8E). Both *Rankl* and *Opg* expressions were high in the osteocyte fractions compared with the osteoblast fractions. However, unloading had no significant effect on the expression of *Rankl* and *Opg* in the osteocyte fractions of both wild-type and *BCL2* transgenic mice at 4 months of age (Fig. 8F).

Wnt antagonists play an important role in bone formation [19], [41], [42]. Thus, we also examined the expression of the genes whose proteins antagonize Wnt signaling in osteocyte fractions (Fig. 8G). *Dkk1* and *sFRP1* expression was high and *sFRP5* expression was low in *BCL2* transgenic mice compared with wild-type mice in the control groups. *Dkk1* was down-regulated after unloading in wild-type mice but not in *BCL2* transgenic mice, and *sFRP5* was down-regulated after unloading in both wild-type and *BCL2* transgenic mice. Unloading had no significant effect on the expression of *Sost*, *sFRP1*, *sFRP2*, and *sFRP4* in both wild-type and *BCL2* transgenic mice at 4 months of age.

### Increase of Sost-positive Osteocytes in the Restricted Region of Tibiae after Unloading in Wild-type Mice but not in *BCL2* Transgenic Mice

The reduction in bone formation in response to unloading is abrogated in *Sost* ^−/−^ mice, and *Sost* mRNA but not Sost-positive osteocytes is increased after unloading [20], [43]. Further, the down-regulation of Sost after loading is dependent on the site in tibiae [44]. Therefore, we examined the expression of Sost on the anterior and posterior sides of cortical bone at the metaphysis and mid-diaphysis of tibiae by immunohistochemistry at 4 months of age (Fig. 9A–D). As the lacunae, which were TUNEL-positive and contained cellular debris of dead osteocytes, were non-specifically reacted with anti-Sost antibody in *BCL2* transgenic mice, we counted only the lacunae containing the cells with a nucleus in both wild-type and *BCL2* transgenic mice. On both sides of cortical bone at metaphysis and the anterior side of cortical bone at mid-diaphysis, the frequencies of Sost-positive cells were similar among wild-type and *BCL2* transgenic mice of both control and unloaded groups (Fig. 9K–M). On the posterior side of cortical bone at mid-diaphysis, however, the frequency was increased in the unloaded group compared with the control group in wild-type mice, while it was similar in both groups in *BCL2* transgenic mice (Fig. 9E–H, N). Further, Sost protein was distributed through the canaliculi throughout bone in wild-type mice but not in *BCL2* transgenic mice probably due to the reduction in the number of osteocyte processes and the accumulation of dead osteocytes in *BCL2* transgenic mice (Fig. 9G–J).

As the distribution of Sost protein was interrupted in *BCL2* transgenic mice at 4 months of age, we compared the protein levels of β-catenin in the osteoblast fractions between wild-type and *BCL2* transgenic mice at 4 months of age by Western blot analysis. β-catenin protein was increased in *BCL2* transgenic mice compared with wild-type mice, suggesting that Wnt signaling was enhanced in osteoblasts of *BCL2* transgenic mice at 4 months of age (Fig. 9O).

### Reduction in Bone Mass in the Unloaded Condition in *BCL2* Transgenic Mice at 6 Weeks of Age

We analyzed the characteristics of osteocytes and the responsiveness to unloading in *BCL2* transgenic mice at 6 weeks of age, when the number of osteocytes was increased but TUNEL-positive lacunae were not accumulated (Figs. 2L, 2M, 3B, 3D). On real-time RT-PCR using RNA from osteocyte-enriched samples at 6 weeks of age, *Dmp1* expression was increased, *Fgf23* expression was decreased, and *Sost*, *Phex*, and *Mepe* were similarly expressed in *BCL2* transgenic mice compared with in wild-type mice (Fig. 10A). Serum levels of calcium and phosphate were similar between wild-type mice and *BCL2* transgenic mice at 6 weeks of age (calcium: wild-type mice 8.58±0.43 mg/dl, *BCL2* transgenic mice 8.66±0.49 mg/dl; phosphate: wild-type mice 11.85±0.79 mg/dl, *BCL2* transgenic mice 11.24±0.9 mg/dl, n  = 5–8). In the unloaded condition, bone volume and trabecular thickness were reduced in the femurs of both wild-type mice and *BCL2* transgenic mice at a similar degree (Fig. 10B, C). After unloading, *Rankl* expression in osteoblasts was marginally increased in both wild-type mice and *BCL2* transgenic mice with less response in the latter at 6 weeks of age. *Opg* expression in osteoblasts was similar between wild-type mice and *BCL2* transgenic mice, and was not affected by unloading (Fig. 10D). The *Rankl* expression in osteocytes was reduced after unloading for 3days in both wild-type and *BCL2* transgenic mice, while unloading had no effect on *Opg* expression in osteocytes (Fig. 10E). *Sost* expression in osteocytes was upregulated after unloading significantly in wild-type mice and marginally in *BCL2* transgenic mice at 6 weeks of age (Fig. 10E).

## Discussion

Unexpectedly, overexpression of *BCL2* in osteoblasts led to the reduction in the number of osteocyte processes, which seemed to be one of the causes of osteocyte apoptosis in *BCL2* transgenic mice [31]. Thus, we examined the effects of osteocyte apoptosis and the reductions in the numbers of osteocytes and their processes on bone formation and resorption using *BCL2* transgenic mice at 4 months of age, when TUNEL-positive lacunae were most accumulated, the number of osteocytes was reduced, and osteocyte network was most severely disturbed, but the expression of transgene was reduced, to minimize the effects of the transgene on osteoblasts and osteocytes. We found that osteoblast function was enhanced and osteoclastogenesis was inhibited in *BCL2* transgenic mice at 4 months of age. These findings suggest that disruption of the osteocyte network might be related to the enhancement of osteoblast function and the suppression of osteoclastogenesis. In the unloaded condition, osteoblast function was inhibited and osteoclastogenesis was enhanced, leading to bone loss in wild-type mice, whereas osteoblast function and osteoclastogenesis were unaffected, leading to the maintenance of bone mass in *BCL2* transgenic mice at 4 months of age. Further, Sost expression in osteocytes and *Rankl* expression in osteoblasts were upregulated in wild-type mice but not in *BCL2* transgenic mice in the unloaded condition. Thus, we propose that the osteocyte network inhibits osteoblast function and stimulates osteoclastogenesis in the physiological condition, and that osteocytes further augment the inhibitory effects on osteoblast function through the induction of Sost in osteocytes and the stimulatory effect on osteoclastogenesis through the induction of *Rankl* in osteoblasts in the unloaded condition (Fig. 11).

In contrast to the general consensus that osteocyte death triggers bone remodeling by enhancing bone resorption [8], [21], [22], osteoclastogenesis was enhanced in parallel with an increase in osteocyte density, and osteoclastogenesis was reduced in parallel with the accumulation of dead osteocytes; therefore, our findings suggest that osteocytes stimulate osteoclastogenesis in the physiological condition. The enhanced bone resorption after osteocyte death in previous observations may have been caused by the stimulation of osteoclastogenesis through an inflammatory reaction in the microenvironment, which would be elicited by the elimination of inflammation-inducible molecules from lacunae through canaliculi when necrotic aspects appear in the cell death of osteocytes that are not phagocytosed [26]. In *BCL2* transgenic mice, however, the reduction in the number of canaliculi and the gradual accumulation of TUNEL-positive lacunae may have limited the release of the inflammation-inducible molecules from lacunae through canaliculi after secondary necrosis, as shown in the immunostaining of Sost (Fig. 9H, J).

We pursued the mechanism of the enhanced bone resorption in the unloaded condition. *Rankl* and *Opg* were highly expressed in osteocyte fractions. Although the upregulation of *Rankl* expression in osteocytes at unloaded condition was reported [29], both *Rankl* and *Opg* expressions in osteocytes were unaffected by unloading. In contrast, *Rankl* but not *Opg* expression was upregulated in osteoblast fractions by unloading in wild-type mice but not in *BCL2* transgenic mice. These findings suggest that the osteocyte network senses unloading and transfers unknown signals to osteoblasts to induce *Rankl* expression in osteoblasts. We found that pyruvate dehydrogenase kinase 4 (Pdk4), which is a negative regulator of pyruvate dehydrogenase complex, is upregulated at unloading and is one of the molecules that induce *Rankl* expression in osteoblasts at the unloaded condition [33]. As soluble factors released from MLO-Y4 regulate osteoclastogenesis [45], [46], osteocytes may release soluble factors to upregulate the expression of *Pdk4* and *Rankl* in osteoblasts. As our findings suggest that the osteocyte network stimulates osteoclastogenesis in the physiological condition, Opg, which was highly expressed in osteocytes, may be trapped by Rankl on the surface of osteocytes. The conditional deletion of *Rankl* using *Dmp1* promoter-Cre transgenic mice has been shown to result in the reduction in bone resorption and increase in bone mass [30], [47]. It may indicate that Opg, which was highly expressed in osteocytes, is secreted to the bone surface through canaliculi in the absence of Rankl on the surface of osteocytes.

The function of osteocytes in bone formation in the physiological condition has been controversial. Acute death of osteocytes by diphtheria toxin severely reduces bone formation [8], while osteocyte density is negatively correlated with bone formation [10], [11]. As both empty lacunar density and periosteal bone apposition increase with age, a link between the two phenomena has been suggested [48], [49], [50], [51], [52]. Furthermore, mice carrying a targeted mutation of *Col1a1*, encoding a collagenase-resistant form of type I collagen, showed osteocyte apoptosis and increased bone formation [53], and it has been shown that osteocytes secrete Sost, which is a potent antagonist of Wnt, and inhibits bone formation [54], [55], [56], [57]; therefore, there were contrasting observations that osteocytes can enhance or inhibit bone formation. Bone formation in both trabecular and cortical bones was enhanced in *BCL2* transgenic mice at 4 months of age when the transgene expression was low, TUNEL-positive lacunae were most accumulated, osteocyte number was reduced, and osteocyte network was disturbed in the whole area of cortical bone. Although we cannot exclude the possibility that the expression of the transgene at the low level promoted bone formation at 4 months of age, this seemed to be unlikely because overexpression of *BCL2* impaired osteoblast differentiation in a manner dependent on the expression levels of the transgene in vivo and in vitro (Fig. 1A) [31], osteoblast density was similar between *BCL2* transgenic mice and wild-type mice at 4 months of age, and cortical bone in *BCL2* transgenic mice was not further increased at 6 months of age irrespective of the similar level of transgene expression at 4 and 6 months of age. The disturbed osteocyte network was gradually restored after 4 months of age, probably because the level of the transgene expression was not sufficient to reduce the number of osteocyte processes. These seemed to be the reasons why the enhanced bone formation was observed only at 4 months of age. Thus, our findings suggest that the reductions in the numbers of osteocytes and their processes and the accumulation of TUNEL-positive lacunae were followed by the activation of osteoblast function, leading to an increase in bone formation. As the distribution of Sost protein was interrupted in osteocytes and β-catenin protein was increased in osteoblasts in *BCL2* transgenic mice at 4 months of age, the activation of Wnt signaling in osteoblasts by the reduction of disseminated Sost protein seems to be one of the causes for the increase in bone formation in *BCL2* transgenic mice at 4 months of age. In mice with osteocyte ablation by diphtheria toxin, the suppression of bone formation seemed to be due to maturational inhibition of osteoblasts, which was shown by the reduction in osteocalcin expression [8], and maturational inhibition may have been caused by a necrosis-induced inflammatory reaction.

The frequencies of TUNEL-positive lacunae in the trabecular bone of *BCL2* transgenic mice were less than those in the cortical bone, probably because trabecular bone is more extensively remodeled than cortical bone and the dead osteocytes in the trabecular bone are rapidly replaced with live osteocytes. Irrespective of the relatively low frequency of TUNEL-positive lacunae, however, the increase of bone formation and unresponsive to unloading were observed in the trabecular bone at 4 months of age. Therefore, the reduction in the number of osteocyte processes in addition to the reduction in the number of osteocytes may be sufficient for the disturbance of osteocyte network in trabecular bone. The number of osteoclasts was reduced in the cortical bone but not in the trabecular bone of *BCL2* transgenic mice at 4 months of age. It may indicate that osteoclastogenesis in cortical bone is more dependent on the osteocyte network than that in trabecular bone, because the number of osteoclasts was already reduced in the cortical bone in *BCL2* transgenic mice at 5–6 weeks of age, when the frequency of TUNEL-positive lacunae was about 20%, an equivalent value detected in the trabecular bone at 4 months of age. The augmented function of the osteocyte network by unloading may be required for the enhancement of osteoclastogenesis by the osteocyte network in trabecular bone.

Our model also may explain why exercise increases bone mass, which has been indicated by many clinical studies [58], because osteocytes decrease the inhibitory effects on bone mass by reducing the inhibitory effect on osteoblast function and the stimulatory effect on osteoclastogenesis in the loaded condition (physiological condition) compared with the unloaded condition (Fig. 11). Our findings suggest that the osteocyte network is a mechanosensor and mechanotransduction system that reduces its negative effects on bone mass by responding to mechanical stress, explaining how bone mass increases with exercise and decreases with bed rest. Our BCL2 transgenic mice was also a useful tool to search the molecular targets of disuse osteoporosis, because we found that Pdk4 is responsible for bone loss at unloading by comparing the genes induced in wild-type mice and BCL2 transgenic mice at 4 months of age in the unloaded condition [33]. Our findings will provide a basis for understanding the osteocyte network, which plays an important role in the regulation of bone mass.

## Supporting Information

Figure S1 **SEM images of endosteum.** SEM images of the endosteum before (A, C) and after (B, D) blushing with a micro-intertooth brush. The boxed regions in A and B are magnified in C and D, respectively. Scale bars = 0.5 mm (A, B); 100 µm (C, D).
(TIF)

Figure S2 **Decrease of osteoid in** ***BCL2*** **transgenic mice with high expression at 10 weeks of age.** Cortical bone (A–D) and trabecular bone (E–H) of femurs in wild-type (A, C, E, G) and *BCL2* transgenic (B, D, F, H) mice with high expression at 10 weeks of age. The boxed regions in A, B, E, and F are magnified in C, D, G, and H, respectively. Osteoid was visualized by Goland-Yoshilki method. Scale bars = 50 µm (A, B, E, F); 10 µm (C, D, G, H). (I) Osteoid thickness. Data are presented as the mean ± S.D. *vs. wild-type mice. **P<0.01, ***P<0.001. wt, 5 mice; tg, 4 mice.
(TIF)

Figure S3 **Canalicular staining (1).** Canalicular staining of femurs at 10 weeks (A, B) and 8 months (E, F) of age and tibiae at 4 months of age (C, D) from wild-type (A, C, E) and *BCL2* transgenic mice (B, D, F). Bone canalicular staining (silver impregnation staining) was performed as previously described [31]. Scale bars = 1 mm.
(TIF)

Figure S4 **Canalicular staining (2).** The boxed anteroproximal regions indicated by “a” in A–F in supplementary figure 3 were magnified in A–F, respectively, in this figure. Scale bars = 100 µm.
(TIF)

Figure S5 **Canalicular staining (3).** The boxed regions in posterior mid-shafts indicated by “b” in A–F in supplementary figure 3 were magnified in A–F, respectively, in this figure. Scale bars = 100 µm.
(TIF)

Figure S6 **Canalicular staining of trabecular bone.** Trabecular bones in wild-type and BCL2 transgenic mice at 4 months of age are shown. Scale bars = 20 µm.
(TIF)
