# Supplementary figures and images for: Osteocyte Network; a Negative Regulatory System for Bone Mass Augmented by the Induction of Rankl in Osteoblasts and Sost in Osteocytes at Unloading
Source: PLoS One. 2012 Jun 29;7(6):e40143. doi: 10.1371/journal.pone.0040143 (PMC3387151; doi:10.1371/journal.pone.0040143)

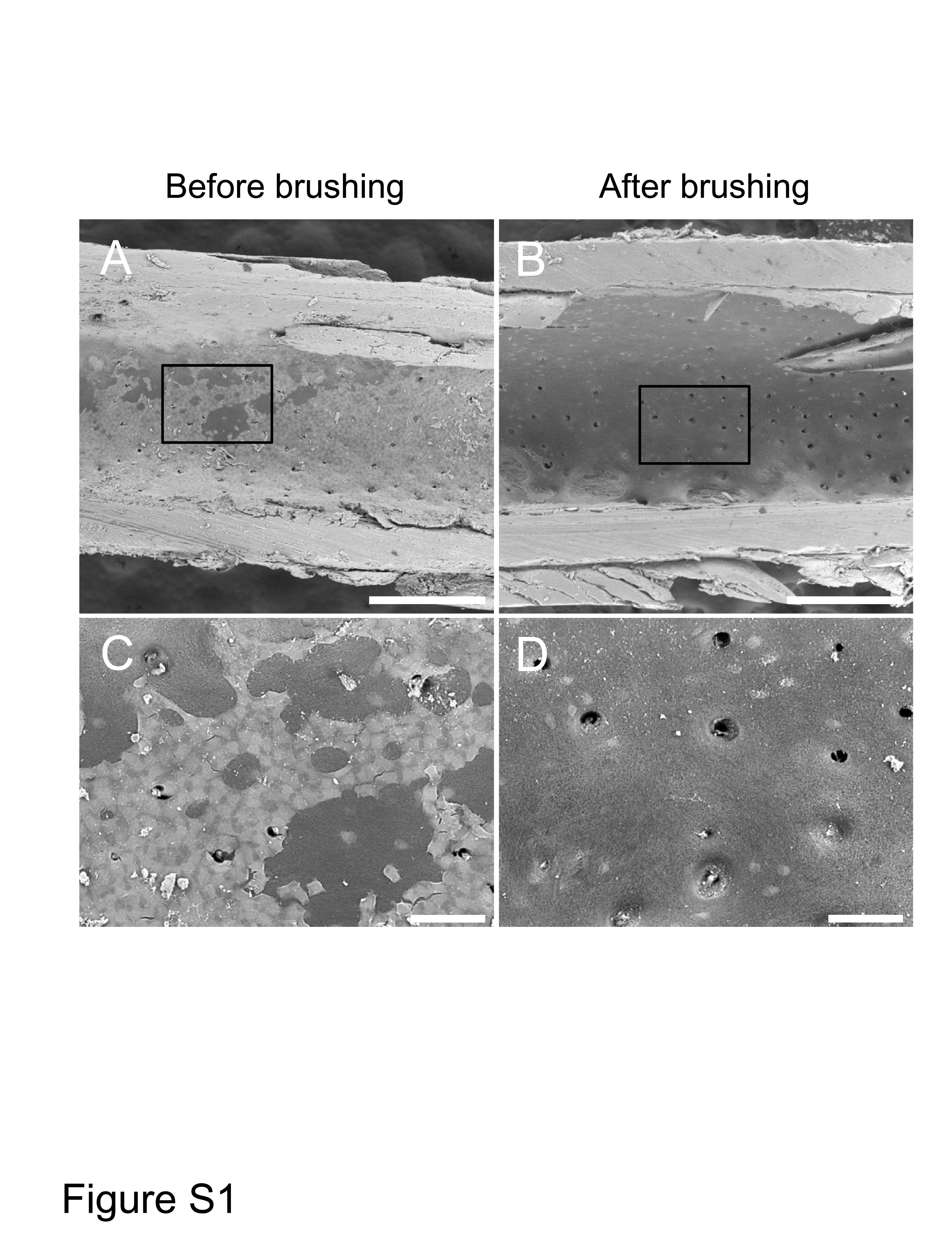

Supplement: Figure S1 — SEM images of endosteum. SEM images of the endosteum before (A, C) and after (B, D) blushing with a micro-intertooth brush. The boxed regions in A and B are magnified in C and D, respectively. Scale bars = 0.5 mm (A, B); 100 µm (C, D). (TIF) [file pone.0040143.s001.tif]

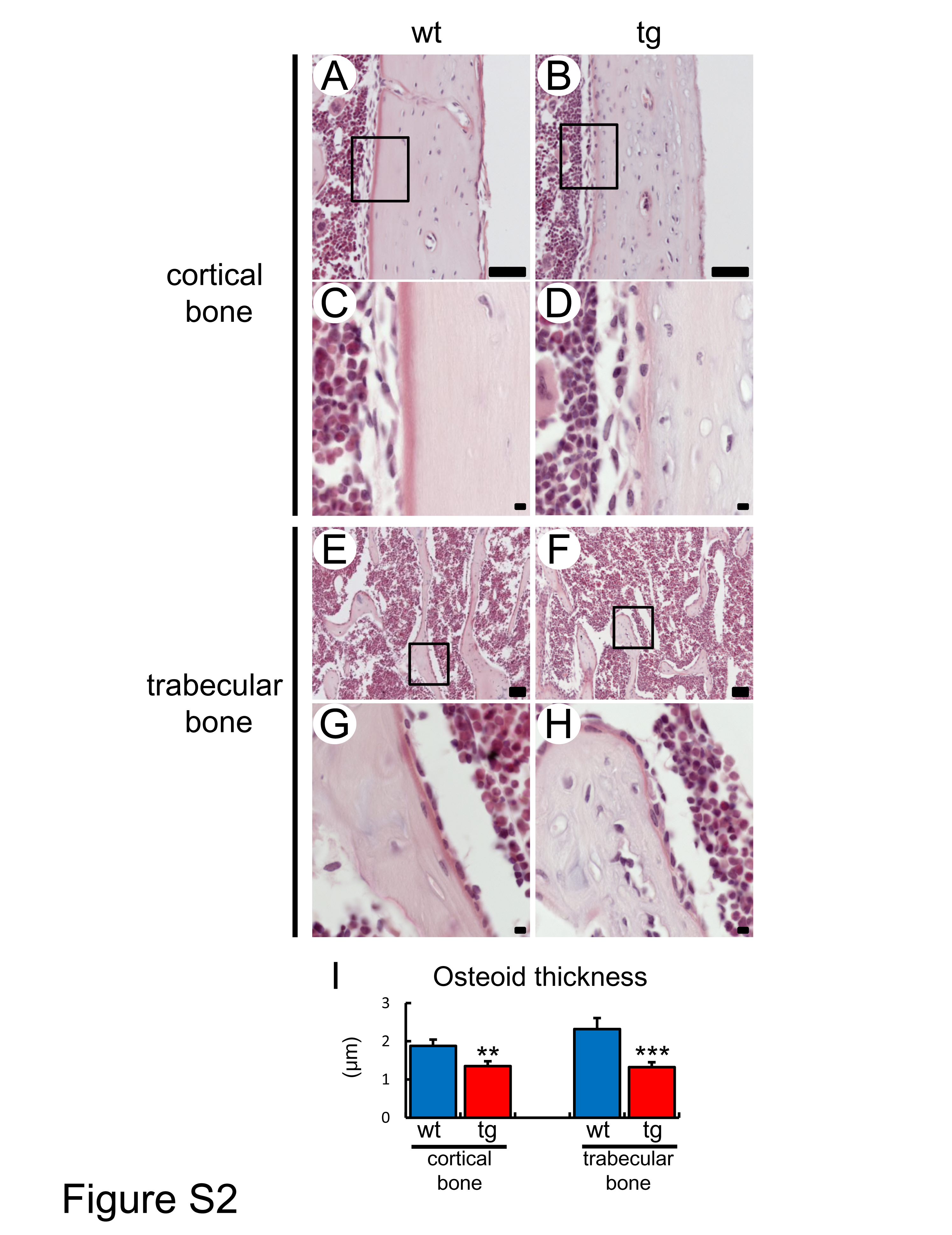

Supplement: Figure S2 — Decrease of osteoid in BCL2 transgenic mice with high expression at 10 weeks of age. Cortical bone (A–D) and trabecular bone (E–H) of femurs in wild-type (A, C, E, G) and BCL2 transgenic (B, D, F, H) mice with high expression at 10 weeks of age. The boxed regions in A, B, E, and F are magnified in C, D, G, and H, respectively. Osteoid was visualized by Goland-Yoshilki method. Scale bars = 50 µm (A, B, E, F); 10 µm (C, D, G, H). (I) Osteoid thickness. Data are presented as the mean ± S.D. *vs. wild-type mice. **P<0.01, ***P<0.001. wt, 5 mice; tg, 4 mice. (TIF) [file pone.0040143.s002.tif]

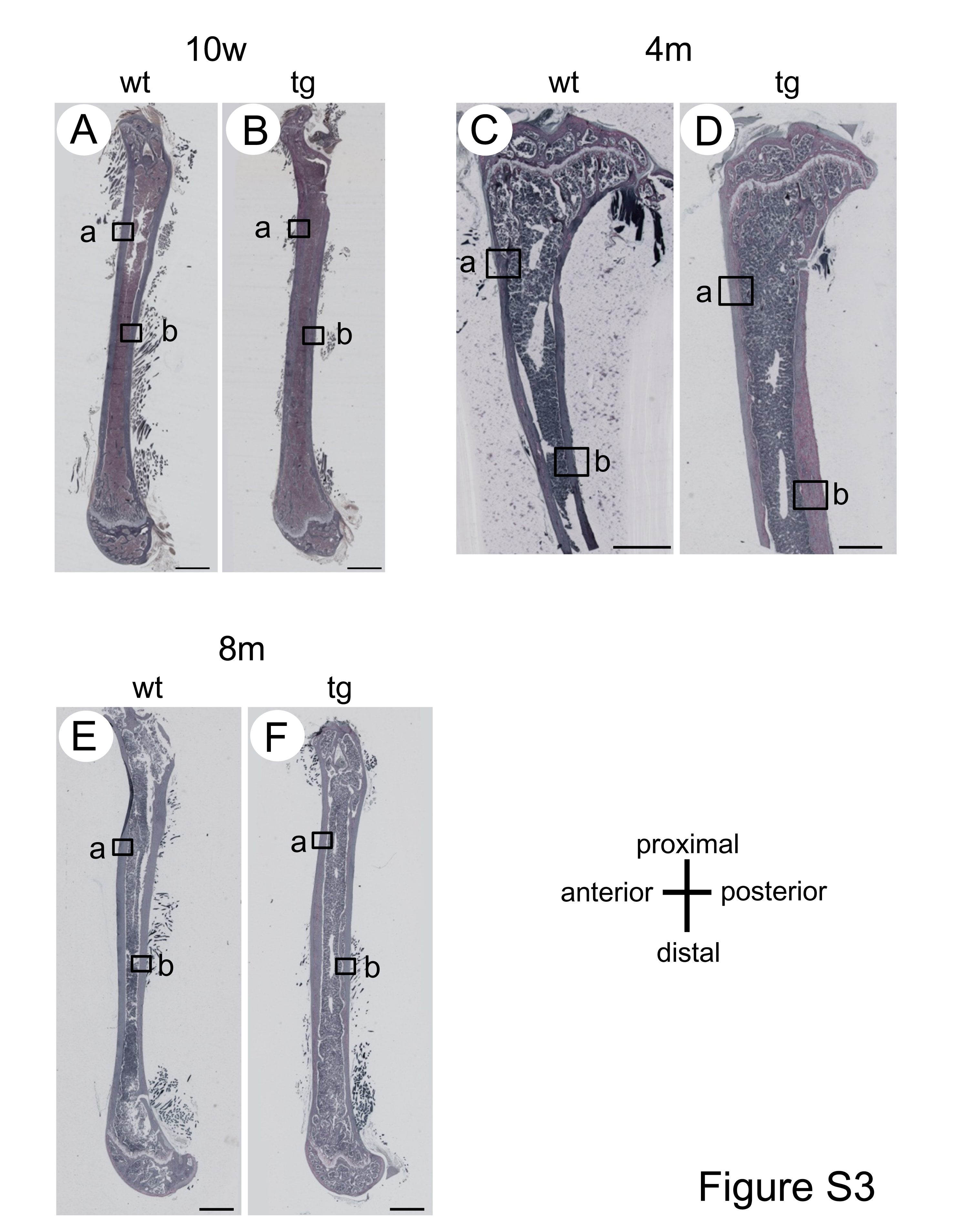

Supplement: Figure S3 — Canalicular staining (1). Canalicular staining of femurs at 10 weeks (A, B) and 8 months (E, F) of age and tibiae at 4 months of age (C, D) from wild-type (A, C, E) and BCL2 transgenic mice (B, D, F). Bone canalicular staining (silver impregnation staining) was performed as previously described [31]. Scale bars = 1 mm. (TIF) [file pone.0040143.s003.tif]

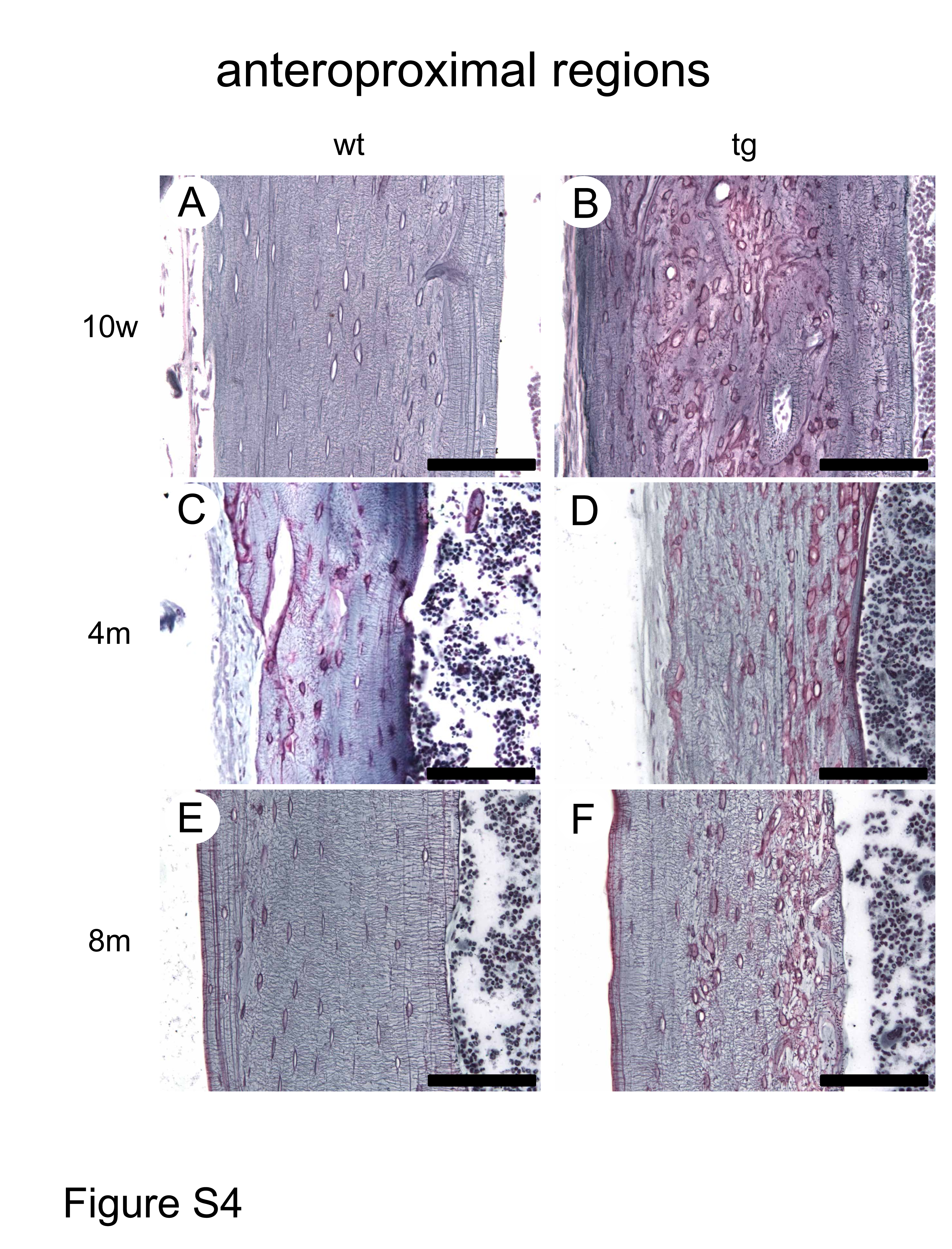

Supplement: Figure S4 — Canalicular staining (2). The boxed anteroproximal regions indicated by “a” in A–F in supplementary figure 3 were magnified in A–F, respectively, in this figure. Scale bars = 100 µm. (TIF) [file pone.0040143.s004.tif]

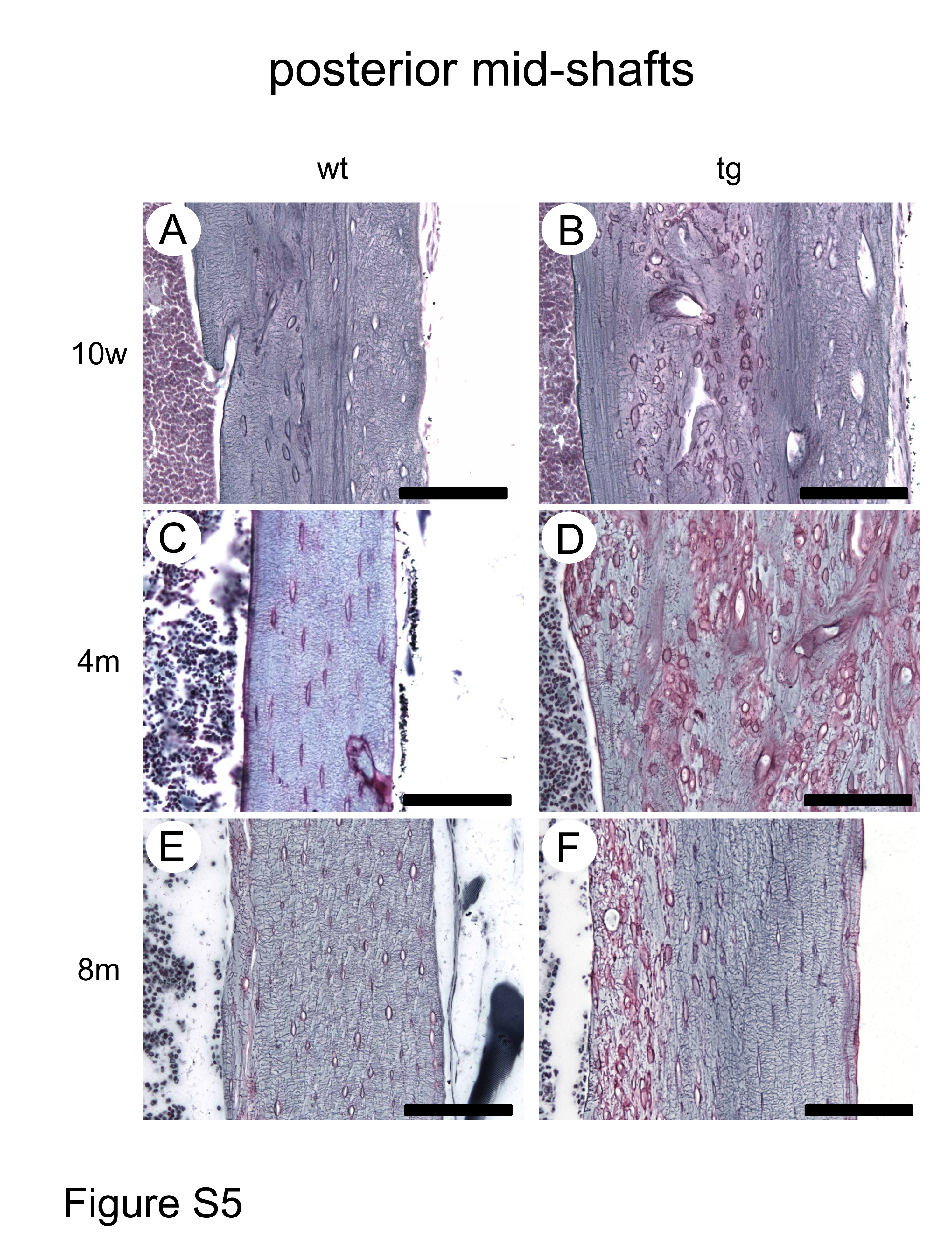

Supplement: Figure S5 — Canalicular staining (3). The boxed regions in posterior mid-shafts indicated by “b” in A–F in supplementary figure 3 were magnified in A–F, respectively, in this figure. Scale bars = 100 µm. (TIF) [file pone.0040143.s005.tif]

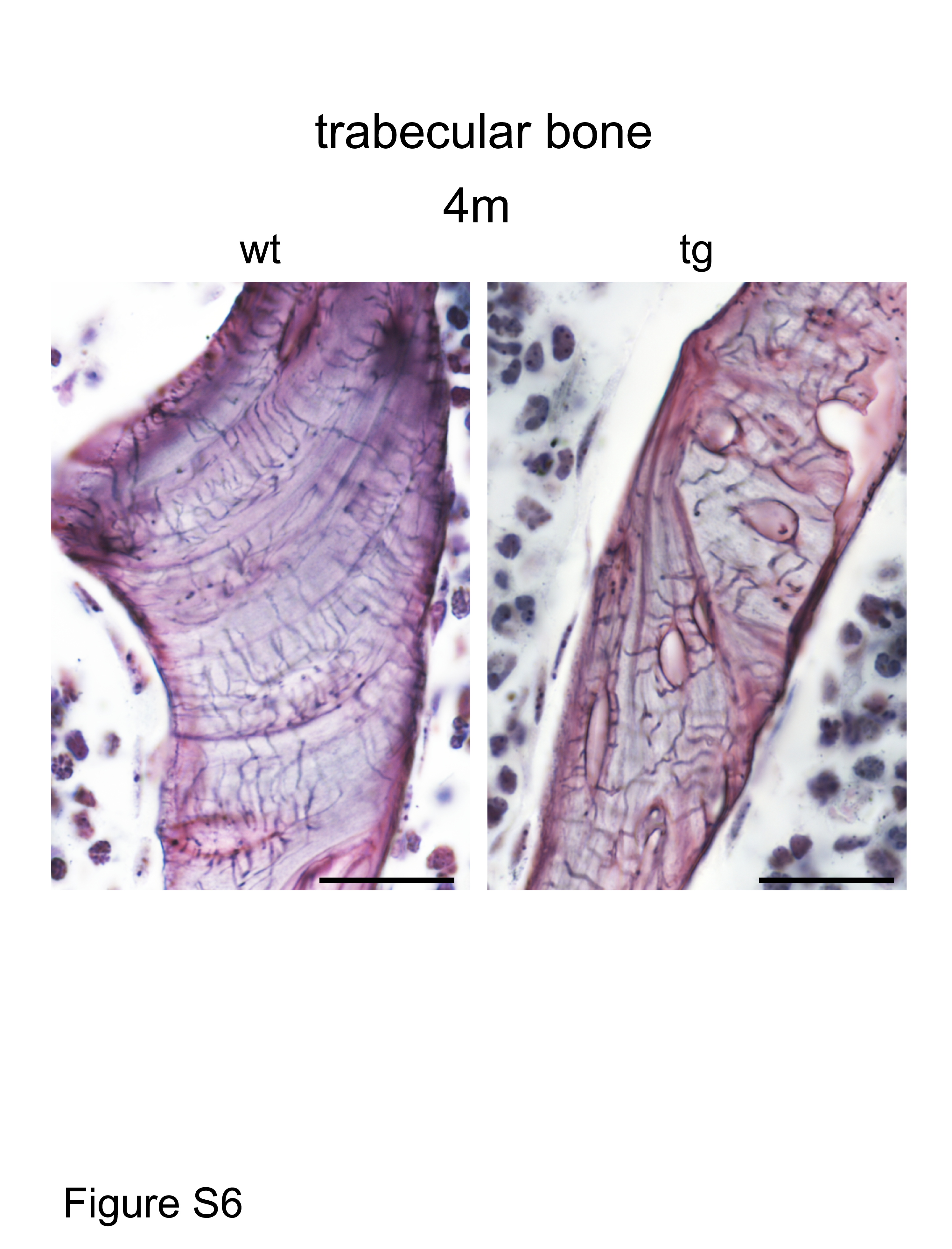

Supplement: Figure S6 — Canalicular staining of trabecular bone. Trabecular bones in wild-type and BCL2 transgenic mice at 4 months of age are shown. Scale bars = 20 µm. (TIF) [file pone.0040143.s006.tif]
